# Targeted Therapy and Immunotherapies in Hepatocellular Carcinoma: Mechanisms and Clinical Studies

**DOI:** 10.1002/mco2.70694

**Published:** 2026-04-20

**Authors:** Penghui He, Sinan Xie, Kunlin Xie, Fengwei Gao, Yunshi Cai, Yan Huang, Chang Liu, Hong Wu, Yinghao Lyu, Tian Lan

**Affiliations:** ^1^ Department of General Surgery West China Hospital Sichuan University Chengdu China; ^2^ Liver Transplant Center Transplant Center West China Hospital Sichuan University Chengdu China; ^3^ Laboratory of Hepatic AI Translation Frontiers Science Center For Disease‐Related Molecular Network West China Hospital Sichuan University Chengdu China; ^4^ Intervention Center West China Hospital Sichuan University Chengdu Sichuan China

**Keywords:** clinical study, hepatocellular carcinoma, immunotherapy, mechanism, targeted therapy

## Abstract

Liver cancer ranks the second leading cause of cancer‐related mortality globally, with a 5‐year overall survival rate of about 14.0%. And hepatocellular carcinoma (HCC) constitutes about 80% of it. Given that HCC often remains asymptomatic in its early stages, the diagnosis of the majority of patients occurs at the intermediate or advanced stages, leading to the missed opportunity for surgical resection. Furthermore, the incidence of recurrence after surgical resection for early‐stage HCC patients can be as high as 70%. In this context, systemic therapies, including targeted therapies and immunotherapies, have emerged as essential therapeutic strategies for advanced HCC. However, resistance and adverse effects limit their efficacy. This review provides an overview of the molecular signaling pathways underlying HCC and potential mechanisms underlying resistance to systemic therapies and discusses potential therapeutic targets within these pathways. We also examine the clinical outcomes of these systemic therapies, highlighting their efficacy, safety profiles, and the challenges of resistance and toxicity. Finally, we outline future directions for HCC treatment, including combination therapies and personalized treatment strategies, which may offer improved treatment outcomes for individuals with HCC.

## Graphical abstract

The mechanism of multiple signaling pathways and their related targeted inhibitors in HCC. (A) The mechanism of Ras/Raf/MEK/ERK signaling pathway and its related targeted inhibitors; (B) the mechanism of PI3K/AKT/mTOR signaling pathway and its crosstalk with other pathways and its related targeted inhibitors; (C) the mechanism of JAK/STAT signaling pathway and its related targeted inhibitors; (D) the mechanism of Wnt/β‐catenin signaling pathway and its related targeted inhibitors; (E) the mechanism of TGF‐β signaling pathway and its related targeted inhibitors; (F) the mechanism of Hh signaling pathway and its crosstalk with other pathways and its related targeted inhibitors.

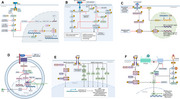

## 1 Introduction

Liver cancer is the second leading cause of cancer‐related fatalities worldwide, with hepatocellular carcinoma (HCC) being the most common subtype, responsible for 75–85% of cases [1]. Owing to the asymptomatic nature of early‐stage HCC, the majority of patients are diagnosed at later stages, either intermediate or advanced, resulting in missed opportunities for curative treatments, including radical resection and liver transplantation. Even for patients who have undergone surgical resection, recurrence occurs in more than half of the them, mostly within 2 years [2, 3]. In this setting, systemic therapy emerged as an innovative treatment approach to prolong survival for advanced HCC patients.

In 2007, sorafenib was authorized by the United States Food and Drug Administration (US FDA) and the European Medicines Agency as the first tyrosine kinase inhibitor (TKI) for advanced HCC. The approval of lenvatinib as a noninferior alternative in the REFLECT trial, followed by subsequent agents including regorafenib, cabozantinib, and ramucirumab, gradually expanded the therapeutic landscape. These targeted therapies, primarily targeting angiogenic and proliferative pathways, have improved outcomes for subsets of patients but are frequently limited by resistance and adverse effects, highlighting the need for novel treatment modalities with more durable efficacy.

In parallel, the advent of immunotherapy has reshaped cancer treatment paradigms. Immune checkpoint inhibitors (ICI) targeting programmed cell death 1 (PD‐1)/programmed cell death ligand 1 (PD‐L1) and cytotoxic T lymphocyte‐associated antigen‐4 (CTLA‐4) have demonstrated meaningful clinical activity in HCC, with durable responses in a subset of patients. Importantly, clinical evidence now validates the application of immunotherapeutic combination regimens in the first‐line setting. The IMbrave150 study established atezolizumab plus bevacizumab as a new standard [4], while the HIMALAYA trial confirmed the benefit of dual checkpoint blockade with tremelimumab and durvalumab [5]. Together, these landmark studies underscore the potential synergy between antiangiogenic therapy and immune modulation and support rational combinations as a key direction for future research.

Despite these advances, overall benefit remains limited by primary and acquired resistance, suboptimal patient selection, and treatment‐related toxicities. Therefore, a thorough understanding of HCC pathogenesis and the clinical results of various systemic treatment options is crucial, as it will provide clinicians with insights to overcome the current barriers in systemic therapy. In the present work, the molecular basis of HCC and its therapeutic targets are first summarized. We then discuss the development of targeted agents, followed by recent progress in immunotherapies and their combinations. Finally, we highlight the importance of predictive biomarkers, challenges in patient selection, and future perspectives. By integrating mechanistic understanding with clinical trial data, this review aims to provide an updated overview for both clinicians and researchers engaged in the management of HCC.

## 2 Molecular Mechanisms in HCC

HCC generally develops in the context of fibrosis or cirrhosis caused by chronic liver disease, including hepatitis B virus (HBV)/hepatitis C virus (HCV) infection, alcohol‐related liver disease, and nonalcoholic fatty liver disease [6, 7, 8]. Its molecular pathogenesis is defined by parallel, highly interactive signaling programs. Pathways such as Wnt/β‐catenin, transforming growth factor‐beta (TGF‐β), and Notch/Hedgehog (Hh) drive de‐differentiation and epithelial–mesenchymal transition (EMT) [9, 10, 11]; growth and metabolic cascades including Ras/Raf/mitogen‐activated protein kinase kinase (MEK)/extracellular‐signal regulated kinase (ERK) and phosphoinositide 3‐kinase (PI3K)/protein kinase B (AKT)/mechanistic target of rapamycin (mTOR) promote proliferation and survival [12, 13]; dysregulation of the Hippo–Yes‐associated protein (YAP) pathway reshapes liver regeneration and tissue‐tension responses [14]; and the Janus‐kinase (JAK)/signal transducer activator of transcription (STAT) and Nuclear Factor kappa‐light‐chain‐enhancer of actived B cells (NF‐κB) p axes translate chronic inflammation and immune disequilibrium into tumor‐promoting signals [15, 16]. These pathways are shaped not only by etiologic heterogeneity but also by extensive interpathway crosstalk, which together dictate tumor sensitivity to targeted therapies, immunotherapies, and their combination and the evolution of resistance. Accordingly, systematically delineating the key signaling circuits and their crosstalk, within the contexts of etiology and the tumor microenvironment (TME), is pivotal for understanding HCC biology and informing precision treatment strategies.

### 2.1 Ras/Raf/MEK/ERK Signaling Pathway

The Ras/Raf/MEK/ERK signaling pathway is one of the most critical and well‐characterized intracellular signaling cascades engaged in the regulation of cell growth, differentiation, survival, and angiogenesis [17]. It is typically activated through the dysregulated overexpression of growth factors and their receptors and the oncogenic mutations in the Ras gene.

**FIGURE 1:**
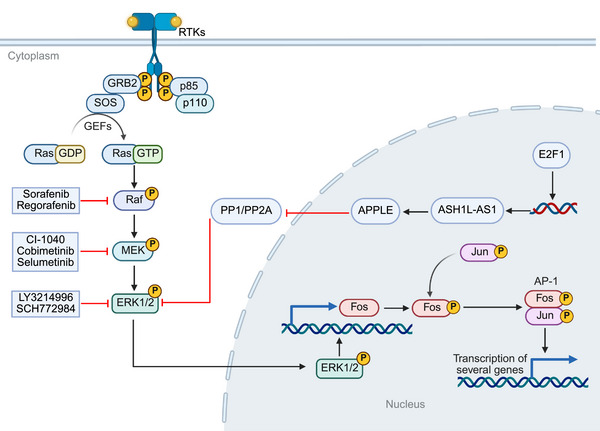
The mechanism of Ras/Raf/MEK/ERK signaling pathway and its related targeted inhibitors. *Abbreviations*: RTK, receptor tyrosine kinase; GRB2, growth factor receptor‐bound protein 2; SOS, son of sevenless; GEF, guanine nucleotide exchange factor; GDP, guanosine diphosphate; GTP, guanosine triphosphate; MEK, mitogen‐activated protein kinase kinase; ERK, extracellular signal‐regulated kinase; PP1, protein phosphatase 1; AP‐1, activator protein 1.

The overexpression of either extracellular ligands, such as epidermal growth factor (EGF), fibroblast growth factor (FGF), platelet‐derived growth factor (PDGF), vascular endothelial growth factor (VEGF), insulin‐like growth factor (IGF), hepatocyte growth factor, and the stem cell growth factor, or their receptor tyrosine kinases (RTKs) could lead to an increased interaction of these ligands with their receptors, which triggers the dimerization and autophosphorylation of RTKs. These phosphorylated RTKs recruit the growth factor receptor‐bound protein 2, which engage the guanine nucleotide exchange factor (GEF) to load guanosine triphosphate (GTP) onto Ras. GTP‐Ras activates Raf, which phosphorylates MEK1/2 and in turn ERK1/2. Activated ERK translocates to the nucleus to stimulate related transcriptional programs, promoting proliferation, apoptosis resistance, metastasis, and angiogenesis (Figure 1) [17]. Approved targeted therapies for HCC are primarily categorized into monoclonal antibodies and small‐molecule TKIs. Monoclonal antibodies such as ramucirumab primarily target RTK. While small‐molecule TKIs, including sorafenib and regorafenib, not only target RTKs but also inhibit Raf kinase. In addition, preclinical and early clinical research has explored inhibitors targeting the downstream signaling molecules of the Ras signaling pathway, such as MEK inhibitors including CI‐1040 [18, 19], cobimetinib [20], and selumetinib [21]. However, the insufficient therapeutic effect and resistance to therapy remain significant challenges.

Although activation of the Ras/Raf/MEK/ERK pathway is common in HCC (approximately half of early‐stage HCC patients exhibit upregulated signaling through this pathway, and it is detected in nearly all advanced‐stage HCC patients), mutations in the key components of this pathway are less frequently detected in HCC [22, 23], which suggests that the activation mechanism of this pathway may rely on nonclassical regulatory pathways that have not yet been fully understood. Recently, Zhao et al. found that the long noncoding RNA (lncRNA) ASH1L–AS1 encodes a microprotein, APPLE, which enhances the phosphorylation of ERK1/2 by binding to and inhibiting protein phosphatase 1 (PP1)/PP2A‐mediated dephosphorylation of ERK1/2, thereby sustaining ERK1/2 phosphorylation and continuously activating the Ras/Raf/MEK/ERK signaling pathway [24]. Furthermore, the transcription factor E2F1 upregulates ASH1L–AS1 transcription, forming an E2F1–ASH1L–AS1/APPLE–ERK1/2 signaling axis [24]. This study suggests that APPLE may be implicated as a promising therapeutic target for HCC.

As the most downstream pivotal point in the Ras/Raf/MEK/ERK pathway, it is reasonable to consider ERK as a therapeutic target. Preclinical models and early clinical data have shown that ERK1/2 inhibitors such as ulixertinib, LY3214996, SCH772984, and GDC‐0994 possess notable antitumor effects [25, 26, 27, 28, 29, 30]. However, the tumors included in these early clinical studies, which demonstrated antitumor activity of ERK, did not include HCC. Some preclinical studies have revealed the antitumor activity of ERK1/2 inhibitor in HCC. For instance, LY3214996 has shown promise in HCC cell models by reversing acquired resistance to sorafenib and enhancing antitumor effects when combined with sorafenib [31]. SCH772984 (ERK1/2 inhibitor) demonstrated antineoplastic effects in experimental HCC models [32]. Nevertheless, the Ras/Raf/MEK/ERK pathway exhibits crosstalk with other signaling pathways, such as PI3K/AKT and Wnt/β‐catenin [33, 34]. Direct targeting of ERK1/2 may not comprehensively block tumor progression and could lead to compensatory activation of other pathways, resulting in resistance. Thus, the use of ERK1/2 inhibitors as a monotherapy requires careful consideration. In the future, the coadministration of ERK1/2 inhibitors with other therapeutic agents might serve as a novel treatment for HCC if the safety is manageable.

### 2.2 PI3K/AKT/mTOR Signaling Pathway

**FIGURE 2:**
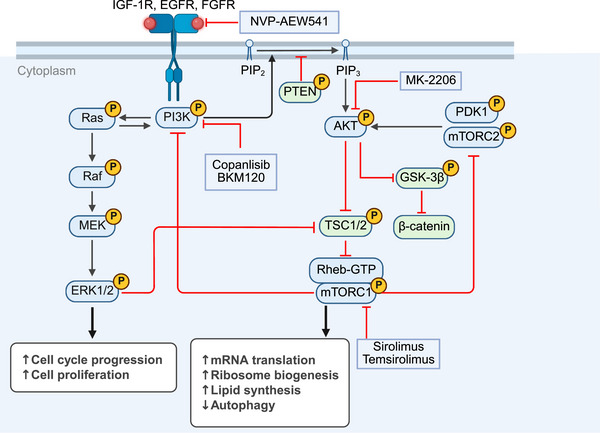
The mechanism of PI3K/AKT/mTOR signaling pathway and its crosstalk with other pathways and its related targeted inhibitors. *Abbreviations*: IGF‐1R, insulin‐like growth factor 1 receptor; EGFR, epidermal growth factor receptor; FGFR, fibroblast growth factor receptor; PI3K, phosphatidylinositol 3‐kinase; PIP2, phosphatidylinositol‐4,5‐bisphosphate; PIP3, phosphatidylinositol‐3,4,5‐diphosphate; AKT, protein kinase B; TSC, tuberous sclerosis complex subunit; Rheb, ras homolog enriched in brain; mTORC1, mammalian target of rapamycin complex 1; PDK1, 3‐phosphoinositide‐dependent protein kinase 1; GSK‐3β; glycogen synthase kinase‐3 beta; MEK, mitogen‐activated protein kinase kinase; ERK, extracellular signal‐regulated kinase.

The PI3K/AKT/mTOR signaling pathway is a critical intracellular cascade, and its dysregulation has been demonstrated to participate in the development of numerous diseases, particularly malignancies [13]. Researches have shown that this pathway is present in about 50% of HCC patients [35]. In the setting of chronic hepatitis, cirrhosis, and hypoxic–angiogenic microenvironment, RTKs such as IGF‐1R, EGFR, and FGFR are persistently activated, which drives PI3K to generate the second messenger phosphatidylinositol‐3,4,5‐diphosphate (PIP3) in combination with PTEN loss‐of‐function mutations or TSC1/2 deficiency, thereby recruiting PH domain‐containing AKT and 3‐phosphoinositide‐dependent protein kinase 1 (PDK1) to the plasma membrane, where AKT is fully activated via phosphorylation at Thr308 by PDK1 and at Ser473 by mTORC2 [36]. Activated AKT inhibits TSC1/2, enabling Rheb‐GTP accumulation and consequent activation of mTORC1, which in turn promotes tumor growth by phosphorylating ribosomal protein S6 kinase 1 and eIF4E‐binding protein 1 to enhance translation initiation and ribosome biogenesis, activating sterol regulatory element‐binding protein 1/2 to stimulate lipid synthesis for membrane biogenesis and energetic demands, and phosphorylating unc‐51 like autophagy activating kinase 1 to suppress autophagy onset (Figure 2) [37].

The PI3K/AKT/mTOR pathway exhibits extensive crosstalk with Hippo–YAP, Ras/Raf/MEK/ERK, and Wnt/β‐catenin signaling. For example, both ERK and AKT converge on TSC2 [33], and AKT phosphorylates glycogen synthase kinase‐3 beta (GSK‐3β) [38], a negative regulator of β‐catenin, to inhibit its activity, leading to β‐catenin deposition in the cytoplasm and its nuclear translocation. Under targeted therapy pressure, compensatory activation of these crossed pathways is likely to occur, contributing to the development of resistance to drugs such as sorafenib and lenvatinib [39, 40]. Overall, the sustained activation of the PI3K/AKT/mTOR pathway is fundamental for driving proliferation and antiapoptotic mechanisms, making it a potential core target in the management of HCC.

Several clinical and preclinical studies have been conducted to assess the efficacy of PI3K/AKT/mTOR pathway blockade in the management of HCC. Copanlisib, a PI3K inhibitor, has demonstrated antitumor activity in several clinical trials [41, 42, 43]. However, its application in HCC remains at the preclinical stage. Ye et al. found that copanlisib showed potential anti‐HCC activity both in single‐agent format and combined with sorafenib treatment [44]. Kirstein et al. discovered that another PI3K inhibitor, BKM120, reduced tumor growth mainly by inhibiting cell‐cycle progression [45]. In the case of AKT inhibitors, MK‐2206 failed to show effective efficacy in advanced biliary tract cancer and was associated with severe side effects [46]. However, recently, Benichou et al. found that carbohydrate responsive element binding protein (ChREBP), by upregulating the regulatory subunit of PI3K (p85α), activates the PI3K/AKT/mTOR pathway and stimulates liver cancer cell proliferation [47]. In HepG2 and Huh7 hepatoma cell lines with stable ChREBP overexpression, inhibiting ChREBP‐induced PI3K/AKT signaling using MK‐2206 notably reduced the ability of ChREBP to promote cell proliferation in vitro [47]. This suggests that MK‐2206 may be effective in treating HCC patients with specific gene expression profiles. The inhibitors of mTOR have also been investigated for the therapeutic value in HCC. For example, sirolimus has been found to exhibit therapeutic potential against HCC across preclinical models and a Phase II clinical trial [48, 49]. Additionally, some combination therapies have also shown therapeutic efficacy against HCC. Ou et al. found that MK‐2206 combined with NVP–AEW541, an IGFR inhibitor, exhibited a synergistic apoptotic effect in HCC, which could be inhibited by the overexpression of survivin [50]. This suggests that survivin may serve as a biomarker to determine whether this combination therapy is suitable for treating HCC. Other combination therapies, such as sirolimus and bevacizumab [51], as well as temsirolimus and sorafenib [52], have also shown potential in treating HCC. Despite the potential therapeutic effects of PI3K/AKT/mTOR inhibitors in HCC, their limited efficacy and significant side effects have prevented breakthrough progress with monotherapy. Future research should focus on molecular profiling to select appropriate patients, thereby enhancing the targeting and effectiveness of treatments. Additionally, combining these inhibitors with ICIs, antiangiogenic drugs, and other therapies should be explored under the premise of ensuring safety to overcome resistance.

### 2.3 JAK/STAT Signaling Pathway

**FIGURE 3:**
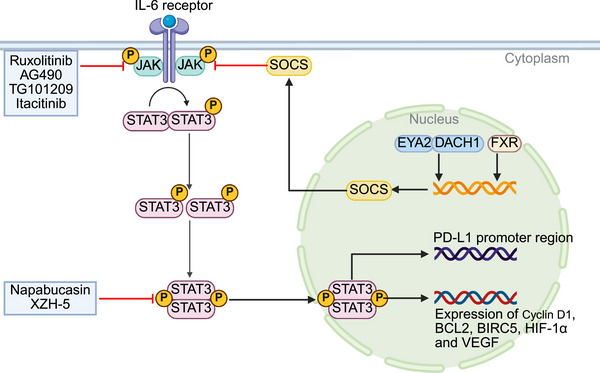
The mechanism of JAK/STAT signaling pathway and its related targeted inhibitors. *Abbreviations*: JAK, Janus kinase; STAT3; signal transducer activator of transcription 3; SOCS, suppressor of cytokine signaling; EYA2, eyes absent homolog 2; DACH1, dachshund homolog 1; RXR, retinoid X receptor; PD‐L1, programmed death‐ligand 1; BCL2, B‐cell lymphoma 2; BIRC5, baculoviral IAP repeat containing 5; HIF‐1α, hypoxia‐inducible factor 1‐alpha; VEGF, vascular endothelial growth factor.

The JAK/STAT signaling pathway is critical for regulating diverse intracellular processes, including immune response, cell survival, and proliferation [15]. In about 50–60% HCC cases, the JAK/STAT pathway is dysregulated, participating in the emergence, progression, and metastasis [53]. The JAK/STAT pathway is mainly composed of three key elements: cytokine receptors, JAKs, and STATs. The pathway is primarily activated by cytokines such as interleukin‐6 (IL‐6), which binds to its cell surface receptor, the IL‐6R. Upon ligand binding, the receptor experiences a structural rearrangement that facilitates the recruitment of JAKs, specifically JAK1 and JAK2. JAKs are then phosphorylated on tyrosine residues, causing a conformational change that activates their kinase activity. Activated JAKs subsequently phosphorylate specific tyrosine residues within the cytoplasmic domain of the IL‐6 receptor, creating docking sites for STATs. The most prominent STAT in HCC is STAT3. The Src‐homology 2 (SH2) domain of STAT3 facilitates its recruitment to the receptor complex, where it interacts with phosphotyrosine residues on the receptor. Once bound, STAT3 undergoes phosphorylation by JAKs, leading to its activation and dimerization. The phosphorylated STAT3 dimers then undergo nuclear translocation and subsequently regulate the expression of specific genes engaged in cell survival, proliferation, angiogenesis and inflammation, which including cyclin D1, B‐cell lymphoma 2, baculoviral IAP repeat containing 5, hypoxia‐inducible factor 1‐alpha (HIF‐1α), and VEGF [54, 55]. Furthermore, STAT3 is known to directly bind to the PD‐L1 promoter region, inducing its transcriptional activation [56]. This activation fosters a proinflammatory TME and supports immune evasion (Figure 3).

In interferon signaling, STAT1 and STAT2 are activated and drive the transcription of antiviral genes, and both STAT1 and STAT2 have been implicated in suppressing HCC cell proliferation [57]. In addition, the JAK/STAT pathway is negatively regulated by suppressors of cytokine signaling 1 (SOCS1) and SOCS3 [54]. These suppressors typically bind to JAKs, block STAT phosphorylation, and promote ubiquitin‐mediated degradation of signaling components, thereby limiting cytokine signaling. However, HCC cells frequently silence SOCS1 or SOCS3 through epigenetic mechanisms such as promoter hypermethylation, thereby relieving this negative feedback [58, 59]. Moreover, SOCS2 was found to prevent liver carcinogenesis through the regulation of STAT5 and STAT3 signaling [60]. Clinically, low expression of SOCS1, SOCS2, and SOCS3 has been observed in HCC and is associated with poor prognosis [61, 62]. Thus, restoring SOCS function, or directly inhibiting JAK/STAT signaling, is considered a potential therapeutic strategy in HCC.

The inhibitors of JAK/STAT signaling primarily include JAK inhibitors and STAT inhibitors. Ruxolitinib, a dual JAK1/2 inhibitor, reduces JAK1 phosphorylation, which subsequently decreases STAT3 activation, thereby inhibiting the proliferation and migration of HCC cells [63]. Other JAK inhibitors, such as AG490 and TG101209, have also demonstrated anti‐HCC effects in preclinical studies [64, 65]. However, the efficacy of these drugs in HCC still requires further clinical validation. For example, a Phase Ib trial investigating the therapeutic value of itacitinib as a second‐line therapy for HCC is currently ongoing [66]. Regarding downstream molecules, STAT3 inhibitors have become a focus of research. Napabucasin, a small molecule STAT3 inhibitor, has demonstrated efficacy in clinical trials for colorectal cancer [67, 68]. Li et al. demonstrated that napabucasin markedly reduced the viability of HCC cells in vitro by triggering apoptosis and inducing cell cycle arrest [69]. Moreover, it exhibited a suppressive effect on HBV replication in HBV‐positive HCC cells [69]. Wang et al. reported that napabucasin inhibits the proliferation of HCC cells and enhances the capacity of the immune system to detect and eradicate tumor cells [70]. Furthermore, the combination of napabucasin with mitoxantrone and PD‐1 inhibitor further suppressed the progression of HCC, inhibited tumor recurrence and prolonged the survival of mice [70]. Liu et al. discovered that XZH‐5, a selective STAT3 inhibitor, reduced sustained phosphorylation of STAT3 at Tyr705 and the expression of STAT3 downstream genes [71]. Moreover, XZH‐5 inhibited IL‐6‐induced STAT3 phosphorylation, nuclear translocation, and DNA binding activity [71]. However, XZH‐5 did not affect IFN‐γ‐induced STAT1 phosphorylation, indicating its more selective effect on STAT3. These results support XZH‐5 as a candidate STAT3‐targeting small molecule with potential applicability in HCC therapy.

Furthermore, since the JAK pathway in HCC is primarily activated by cytokines such as IL‐6 binding to cytokine receptors, cytokines may also serve as a target for cancer therapy. Clazakizumab and siltuximab, two anti‐IL‐6 antibodies, have demonstrated encouraging clinical benefit in clinical studies for the treatment of rheumatoid arthritis and Castleman disease [72, 73]. Wang et al. found that IL‐6 may promote tumor progression by activating the JAK/STAT3 signaling pathway during NVP–BEZ235 (an innovative dual PI3K/mTOR inhibitor) treatment of HCC [74]. The combination of anti‐IL‐6 antibodies enhanced the inhibitory effect of NVP–BEZ235 on the PI3K/AKT/mTOR pathway and significantly improved antitumor efficacy [74]. Additionally, in vivo studies revealed that the coadministration of NVP–BEZ235 and anti‐IL‐6 antibodies was more effective in reducing HCC tumor burden compared with either agent alone [74]. This indicates the crosstalk between signaling pathways and resistance mechanisms during HCC pathogenesis, providing a new strategy for HCC treatment that warrants further exploration in preclinical studies and clinical trials.

Liu et al. found that eyes absent homolog 2 (EYA2) binds to dachshund homolog 1 (DACH1) to transcriptionally regulate SOCS3 expression, which in turn inhibits HCC progression via the SOCS3‐mediated JAK/STAT signaling pathway [75]. Ye et al. discovered that activation of the farnesoid X receptor (FXR) upregulates SOCS3 expression, inhibiting STAT3 phosphorylation and thereby suppressing the malignant phenotype of HCC stem cells [76]. In a diethylnitrosamine‐induced mouse model of HCC, FXR‐deficient mice exhibited a higher incidence of HCC, accompanied by reduced SOCS3 expression and increased STAT3 phosphorylation levels [76]. Recently, Bernal et al. found that a SOCS1 mimic peptide demonstrated hepatoprotective effects in experimental metabolic‐associated fatty liver disease/metabolic‐associated steatohepatitis by regulating lipotoxicity, inflammation, redox balance, and fibrosis [77]. These studies suggest that SOCS deficiency contributes substantially to hepatocyte proliferation, and restoring its function may regulate the proliferation of hepatocytes, offering potential therapeutic significance for HCC treatment.

### 2.4 Wnt/β‐Catenin Signaling Pathway

Aberrant activation of the Wnt/β‐catenin pathway is essential for hepatocarcinogenesis, with abnormal accumulation of β‐catenin in 17–40% of HCC patients [78]. The pathway is composed of three major components: Wnt signaling proteins, the β‐catenin destruction complex, and transcriptional regulators.

**FIGURE 4:**
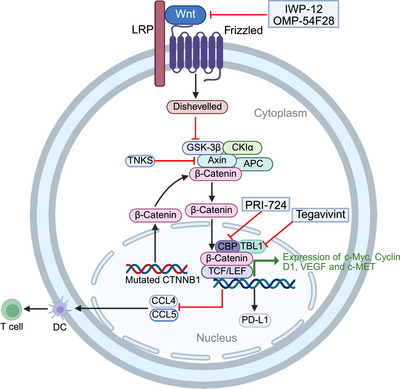
The mechanism of Wnt/β‐catenin signaling pathway and its related targeted inhibitors. *Abbreviations*: LRP, lipoprotein receptor‐related proteins; GSK‐3β, glycogen synthase kinase‐3 beta; CKIa, casein kinase I a; APC, adenomatous polyposis coli; TCF/LEF, T‐cell factor/lymphoid enhancer factor; CBP, CREB‐binding protein; TBL1, transducin beta‐like 1; CCL4, C‐C motif chemokine ligand 4; DC, dendritic cell; VEGF, vascular endothelial growth factor; PD‐L1, programmed death‐ligand 1; TNKS, tankyrase.

Pathway activation begins with ligand–receptor interactions between Wnt proteins and cell‐surface Frizzled receptors. Axin 1, as the main organizer of the degradation complex, carries binding sites for adenomatous polyposis coli (APC), GSK‐3α/β, and casein kinase I a (CKIa), regulating the degradation of β‐catenin. Following the binding of Wnt protein ligands to Frizzled receptors, lipoprotein receptor‐related protein (LRP) plays a coactivating role by facilitating the binding, ultimately forming a ternary complex. Subsequently, the intracellular region of Frizzled facilitates plasma membrane localization of Dishevelled proteins, promoting LRP6 phosphorylation. This, in turn, further recruits Axin and GSK‐3β, inhibiting the activity of the degradation complex and leading to β‐catenin enrichment. The accumulated β‐catenin translocates into the nucleus, thereby engaging T‐cell factor/lymphoid enhancer factor, activating genes such as c‐Myc, cyclin D1, VEGF, and c‐MET, thereby driving cell proliferation and angiogenesis (Figure 4) [79, 80].

On the other hand, the Wnt/β‐catenin pathway also exerts substantial regulatory effects on the HCC TME. Dendritic cells regulate T cell function by recognizing and presenting antigens to T cells. Studies have shown that sustained activation of tumor‐derived β‐catenin can impact antitumor immunity by influencing the recruitment of dendritic cells to the TME [81]. The failure of dendritic cell recruitment is partially attributed to the reduction of C–C chemokine ligand 4 (CCL4) [82, 83], which are induced by β‐catenin activation in tumor cells. In addition, researches have shown that the sparse presence of T cells within tumors in HCC patients is associated with CTNNB1 mutations(encoding β‐catenin) [84, 85]. According to Calderaro et al. [86], HCC could be classified into six types. Among them, the CTNNB1 mutated HCC is featured by lack of immune infiltration. The exclusion of T cells could partially be contributed to the failure of DC recruitment. Moreover, nuclear β‐catenin can also negatively regulate the production of CCL5 [81], which is involved in T cell recruitment (Figure 4).

In parallel, Wnt/β‐catenin pathway promotes immune evasion by elevating PD‐L1 levels in tumor cells [87]. Recently, Shao et al. reported that Wnt/β‐catenin pathway inhibition can reduce PD‐L1 expression in HCC cells by suppressing its translation and enhance the efficacy of PD‐1 blockade therapy [88]. Researchers treated HuH7 and Hep3B cells with two Wnt pathway inhibitors, XAV939 and IWR‐1, and found that these inhibitors significantly reduced PD‐L1 protein expression induced by interferon‐γ in a dose‐dependent manner, while also decreasing the level of active β‐catenin.

The inhibition strategies targeting the Wnt signaling pathway mainly focus on the following key steps: blocking the secretion of Wnt ligands or receptor binding, stabilizing the β‐catenin degradation complex, and directly blocking the interaction between β‐catenin and its transcriptional coactivators. Research has reported that the porcupine (an O‐acyltransferase essential for Wnt ligand secretion and processing) inhibitor IWP‐12, or knockdown of Wntless (a protein essential for Wnt secretion), can reduce Wnt ligand secretion in HCC cell lines, thereby inhibiting cell proliferation [89]. In animal models, treatment with OMP‐54F28(which blocks Wnt signaling by binding to Wnt ligands) in a xenograft model derived from HCC patient samples demonstrated that monotherapy could inhibit tumor growth by approximately 46%, while the combination with sorafenib enhanced the tumor growth inhibition to about 78% [90]. Additionally, studies have also investigated the function of stabilizing the β‐catenin degradation complex in HCC treatment. Tankyrase (TNKS) promotes Axin ubiquitination and proteasomal degradation through adenosine diphosphate ribosylation [91]. It has been found that inhibiting TNKS significantly increases Axin protein levels, thereby enhancing the ability of degradation complex, facilitating β‐catenin phosphorylation and subsequent degradation, ultimately resulting in decreased HCC cell proliferation and reduced colony formation [92]. Considering that the interaction between β‐catenin and its transcriptional coactivators represents a critical downstream event in the Wnt/β‐catenin signaling, and that HCCs with mutations in the CTNNB1 gene may not require upstream signaling to enable nuclear translocation of β‐catenin, blocking β‐catenin–coactivator interaction may present a more promising therapeutic target. A recent study reported that β‐catenin activation correlates with sorafenib resistance in HCC models [93]. Besides, combined treatment with PRI‐724 (a CREB‐binding protein/β‐catenin transcriptional antagonist) and sorafenib resulted in synergistic antitumor activity both in vitro and in vivo [93]. Moreover, in HCC, transducin beta‐like 1 (TBL1) forms a complex with nuclear β‐catenin to promote tumor gene transcription [94]. A Phase I/II clinical trial is ongoing for the TBL1 inhibitor tegavivint (BC2059) among advanced HCC patients harboring activating mutations in β‐catenin (NCT05797805). Targeting the Wnt pathway in HCC therapy has been preliminarily validated, but most approaches are still in the preclinical stage. Future strategies may require combination with existing treatments (e.g., TKIs, immunotherapy) to enhance efficacy. Furthermore, since not all HCC patients exhibit activation of Wnt pathway, biomarkers such as CTNNB1 mutation status and β‐catenin nuclear localization may help identify patient subgroups that are expected to respond to such treatments.

### 2.5 TGF‐β Signaling Pathway

Approximately 40% of HCC patients exhibit genetic mutations in components of the TGF‐β signaling pathway [95]. The TGF‐β signaling pathway plays an extremely complex and bidirectional role in the pathogenesis of HCC, exhibiting both tumor‐suppressive and tumor‐promoting effects [96, 97]. The molecular mechanisms of the TGF‐β signaling pathway can be broadly classified into two categories: the canonical SMAD‐dependent pathway and the SMAD‐independent pathway [98].

#### 2.5.1 The Canonical SMAD‐Dependent Pathway

**FIGURE 5:**
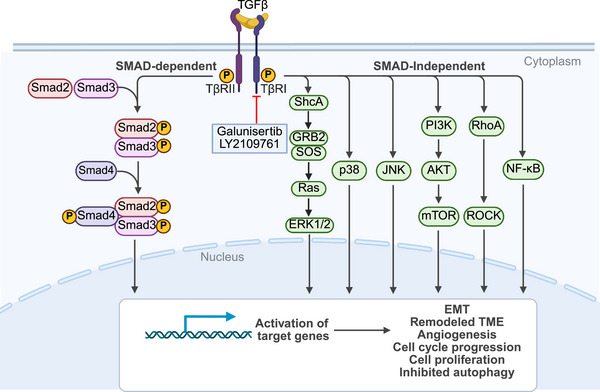
The mechanism of TGF‐β signaling pathway and its related targeted inhibitors. *Abbreviations*: TGF‐β, transforming growth factor beta; TβR, TGF‐β receptor; Smad, signal transducer activator of transcription; ShcA, src homology 2 domain containing adapter protein A; GRB2, growth factor receptor‐bound protein 2; SOS, son of sevenless; ERK, extracellular signal‐regulated kinase; JNK, c‐Jun N‐terminal kinase; PI3K, phosphatidylinositol 3‐kinase; AKT, protein kinase B; mTOR, mammalian target of rapamycin; RhoA; ras homolog family member A; ROCK, rho‐associated protein kinase; NF‐κB, nuclear factor kappa‐light‐chain‐enhancer of activated B cells; EMT, epithelial–mesenchymal transition; TME, tumor microenvironment.

TGF‐β ligands typically exist in the extracellular matrix as inactive complexes, which consist of TGF‐β, latency‐associated peptide (LAP), and latent TGF‐β‐binding protein [99]. Under specific conditions, such as tissue injury or inflammatory responses, plasmin or matrix metalloproteinases (MMPs) cleave LAP from TGF‐β, thereby releasing the active TGF‐β ligand. The active TGF‐β ligand first binds to TGF‐β receptor II (TβRII), a transmembrane serine/threonine kinase receptor that exhibits constitutive kinase activity, meaning it retains a certain level of kinase activity even without ligand interaction. TGF‐β binding to TβRII results in further activation of TβRII kinase activity. Activated TβRII phosphorylates the GS domain (rich in glycine and serine) of TGF‐β receptor I (TβRI), thereby activating TβRI. Once TβRI is activated, TβRI and TβRII form a stable and active receptor complex. The activated TβRI recruits and phosphorylates receptor‐regulated Smads (R‐Smads), primarily Smad2 and Smad3. Phosphorylation induces a conformational change in Smad2 and Smad3, exposing nuclear localization signals (NLS) within their MH1 domains, and they are then able to bind to the common mediator Smad (Co‐Smad), namely, Smad4, to form a complex. The resulting Smad2/3‐Smad4 complex is transported into the nucleus via its NLS. Inside the nucleus, the Smad2/3‐Smad4 complex interacts with other transcription factors to modulate the transcriptional regulation of target genes (Figure 5) [100, 101].

In the early stages of HCC, the canonical TGF‐β/Smad pathway primarily exhibits tumor‐suppressive functions: it inhibits the proliferation and malignant transformation of hepatocytes or preneoplastic hepatocytes by inducing cell cycle arrest (such as upregulation of p15^INK4B, p21^CIP1, inhibition of c‐Myc, and Bcl‐2), promoting apoptosis, autophagy, and cellular senescence [97, 102]. However, as the tumor progresses to advanced stages or the TME undergoes changes, the role of this pathway may switch to a protumorigenic function. Potential mechanisms for this shift include the transition of Smad2/3 C‐terminal phosphorylation to linker region phosphorylation, Smad4 functional impairment, and downregulation of inhibitory Smad expression, all of which lead to the loss of tumor‐suppressive functions of the classical pathway and its conversion to a tumor‐promoting role [97]. These protumorigenic behaviors include: promoting EMT, enhancing invasion/metastasis, inducing tumor stem cell properties, remodeling the TME (such as activating hepatic stellate cells [HSCs], fibrosis, and matrix production), and promoting immune evasion (e.g., Treg recruitment and inhibition of effector T cells) [97, 102].

#### 2.5.2 The SMAD‐Independent Pathway

In addition to the canonical SMAD‐dependent cascade, TGF‐β also engages multiple SMAD‐independent signaling pathways, which collectively diversify its cellular effects and contribute to disease progression.

The SMAD‐independent branch is exemplified by the activation of Ras/Raf/MEK/ERK pathway. Through the activation of ERK, c‐Jun N‐terminal kinase, and related kinases, TGF‐β regulates a broad spectrum of biological processes, such as cell proliferation, apoptosis, and metastasis. Mechanistically, activated TβRI induces tyrosine phosphorylation of src homology 2 domain containing adapter protein A (ShcA), thereby facilitating the assembly of the ShcA/growth factor receptor‐bound protein 2 (GRB2)/son of sevenless (SOS) complex. This sequentially activates Ras and the downstream Raf/MEK/ERK cascade [103, 104]. Activated ERK1/2 phosphorylates transcription factors to drive TGF‐β‐induced transcriptional responses and further modulates Smad activity, underscoring the intricate crosstalk between canonical and noncanonical branches [104].

TGF‐β can also activate the PI3K/AKT axis, either directly via the TβR complex or through tumor necrosis factor receptor‐associated factor 6 (TRAF6)‐mediated ubiquitination of the PI3K regulatory subunit p85α [101, 105]. This pathway is crucial for promoting cell survival, metabolism, proliferation, and differentiation, particularly in the context of tumorigenesis.

Another important noncanonical branch involves the Rho GTPase signaling cascade. TGF‐β rapidly triggers Rho activation in a SMAD‐independent manner [104]. Activated Rho stimulates Rho‐associated protein kinase 1 (ROCK1), which subsequently phosphorylates LIM domain kinase 2 (LIMK2). LIMK2 then phosphorylates cofilin, inhibiting actin depolymerization and driving cytoskeletal reorganization [106]. Beyond cytoskeletal regulation, the Rho/ROCK1 axis enhances ERK phosphorylation, creating synergistic signaling interactions [107, 108]. Functionally, this pathway regulates cell morphology, adhesion, migration, and transcription, and has been strongly implicated in fibrosis and tumor progression.

TGF‐β can also activate the NF‐κB signaling cascade, primarily through TRAF6‐dependent mechanisms. Upon stimulation, TRAF6 undergoes K63‐linked polyubiquitination, which recruits and activates TGF‐β activated kinase 1 (TAK1) [109, 110]. Activated TAK1 then phosphorylates the IκB kinase complex, leading to IκB degradation and subsequent NF‐κB translocation to the nucleus [109]. Nuclear NF‐κB drives the transcription of target genes participated in inflammation, immune regulation, and cell survival [111]. In pathological contexts, such as fibrosis and cancer, aberrant NF‐κB activation by TGF‐β fosters a proinflammatory microenvironment and supports tumor progression [112].

Collectively, these SMAD‐independent pathways provide TGF‐β with versatile regulatory potential beyond its canonical SMAD‐mediated signaling. By activating Ras/Raf/MEK/ERK, PI3K/Akt, Rho GTPase, and NF‐κB cascades, TGF‐β orchestrates complex cellular responses that integrate survival, proliferation, cytoskeletal remodeling, immune modulation, and transcriptional regulation, thereby playing a decisive role in fibrogenesis and cancer development (Figure 5).

In the treatment of HCC, inhibitors targeting the TGF‐β pathway have gradually become a research focus. Preclinical studies have shown that TβRI inhibitors can reduce Smad2 phosphorylation, inhibit invasive and migratory properties of tumor cells, and decrease tumorigenic potential in HCC cell lines. Specifically, in vitro experiments indicated that galunisertib effectively reduces phosphorylated Smad2 levels and inhibits invasion in various HCC cell lines [113]. The TβRI inhibitor LY2109761 has also been shown to significantly suppresses HCC growth, angiogenesis, and metastasis in vivo models [114]. Moreover, TGF‐β inhibition has been utilized in combination therapy strategies. For instance, it was found that TGF‐β inhibition enhances the antitumor activity of targeted therapy and immunotherapy in HCC models [113, 115, 116].

Clinically, it has been found that in patients with HCC who experienced disease progression or intolerance to sorafenib, those with a decrease in circulating alpha‐fetoprotein (AFP) and TGF‐β1 levels after galunisertib treatment exhibited prolonged survival [117]. Additionally, studies have shown that the combination of galunisertib with sorafenib exhibited acceptable tolerance and efficacy in advanced HCC patients [118, 119].

Despite these promising results, TGF‐β pathway inhibitors still face several challenges in HCC treatment. First, given the involvement of TGF‐β in normal liver regeneration, fibrosis, and liver tissue homeostasis, its complete inhibition may pose a risk to liver function. Second, the TGF‐β pathway exhibits a dual function in HCC. Therefore, it is crucial to select appropriate patients, assess the activation status of the pathway, and determine the optimal timing for treatment. Continued research efforts should be directed toward the precise identification of patient subgroups likely to benefit (such as those whose pathway has transitioned from tumor‐suppressive to tumor‐promoting), the establishment of biomarkers (e.g., linker region phosphorylated Smad2/3 levels, AFP, TGF‐β1 expression), and the development of combination immunotherapy/targeted therapy strategies.

### 2.6 Hippo–YAP Signaling Pathway

The Hippo–YAP signaling pathway functions as a core regulator of organ size, tissue regeneration, and stem cell maintenance by controlling cell proliferation, survival, and apoptosis [120, 121]. In the pathophysiology of HCC, the Hippo pathway is frequently dysregulated, resulting in dysregulated activation of its downstream effectors, YAP and transcriptional coactivator with PDZ‐binding motif (TAZ). Under normal conditions, the Hippo–YAP pathway negatively regulates YAP/TAZ by phosphorylating them, which promotes their cytoplasmic sequestration and prevents nuclear import [122]. However, in HCC, mutations in upstream regulators of the Hippo pathway, such as mammalian sterile 20‐like kinase1/2(MST1/2) and large tumor suppressor kinase 1/2(LATS1/2), along with changes in the TME, cause inactivation of the pathway [123]. This results in the dephosphorylation of YAP/TAZ, enabling their nuclear localization and subsequent interaction with transcriptional enhanced associate domain (TEAD) transcription factors to activate genes promoting cell cycle progression and inhibiting apoptosis, providing a growth advantage to HCC cells [124].

In addition, YAP/TAZ also promote tumor metastasis by regulating EMT. They upregulate mesenchymal markers (e.g., vimentin, N‐cadherin, fibronectin) and downregulate epithelial markers (e.g., E‐cadherin), facilitating cell adhesion loss and enhancing tumor cell invasiveness [125]. Besides, YAP/TAZ enhance the secretion of MMPs, aiding tumor cell invasion and metastasis [126]. Moreover, YAP/TAZ activation drives abnormal angiogenesis by upregulating VEGF, angiopoietins, and FGFs, leading to dysfunctional, leaky blood vessels that support tumor growth and metastasis, and integrating with other signaling pathways such as Ras/Raf/MEK/ERK pathway further promote HCC development [121, 127].

Current researches on YAP/TAZ inhibitors for cancer treatment primarily focus on other malignancies, including lung and gastric cancer. In the context of HCC, most studies are still in the preclinical stage. One study found that the ubiquitin‐specific protease 1 (USP1) enhances TAZ stability, thereby promoting HCC progression [128]. Inhibition of USP1 suppresses Hippo/TAZ axis activity and inhibits HCC cell growth and migration [128]. Additionally, another study suggested that the natural product cordycepin upregulates MST1 and LATS1 expression, subsequently inhibiting YAP1 and reducing HCC cell proliferation and migration [129]. Up to this point, no clinical trials have been conducted specifically for Hippo pathway inhibitors in HCC. However, a Phase I clinical trial evaluating a TEAD inhibitor in patients with solid tumors, including HCC, is currently in progress (NCT05228015). Despite this, the application of such inhibitors in HCC faces several challenges. For example, the nuclear localization of YAP/TAZ, TEAD binding activity, and phosphorylation status of upstream MST1/2 and LATS1/2 may vary significantly across different HCC cases. Identifying the patient population suitable for YAP/TAZ inhibition therapy remains a major challenge. Since YAP/TAZ–TEAD is a transcriptional coactivator engaged in a range of physiological processes (including liver regeneration, cell proliferation, and liver tissue homeostasis), inhibitors need to strike a balance between tumor‐killing effects and the preservation of normal liver function.

### 2.7 Notch Signaling Pathway

The notch signaling pathway is a fundamental mechanism of cell–cell communication, which regulates key cellular processes, such as proliferation, differentiation, survival, and apoptosis [130]. In tumors, notch signaling is instrumental in fostering tumorigenesis, metastasis, and angiogenesis [131].

#### 2.7.1 Canonical Notch Signaling

The canonical notch pathway is activated when the notch receptors (notch1–4) on the target cell surface bind to ligands presented on adjacent cells, specifically Jagged1/2 or delta‐like ligands (DLL1, DLL3, DLL4) [130]. Upon ligand binding, the notch receptor undergoes proteolytic cleavage by A Disintegrin and Metalloprotease (ADAM) family enzymes, followed by a γ‐secretase cleavage. This cleavage liberates the notch intracellular domain (NICD) and directs it to the nucleus [130]. In the nucleus, NICD binds to recombination signal binding protein Jκ, also known as CBF1/Su(H)/Lag1 (CSL), and recruits coactivators such as mastermind‐like 1, forming a complex that activates the transcription of target genes [130, 132].

In HCC, the classical notch signaling pathway has been identified as being dysregulated. Activation of notch in HCC is associated with increased tumor aggressiveness, chemoresistance, and poor prognosis [133, 134, 135]. Key target genes activated by notch signaling include cyclin D1, c‐Myc, Hes1, and Hey1, all of which contribute substantially to cell cycle regulation, stemness maintenance, and antiapoptotic processes [130].

#### 2.7.2 Noncanonical Notch Signaling

In addition to the classical notch signaling pathway, noncanonical notch signaling refers to ligand‐independent mechanisms through which notch receptors influence cellular behavior. The notch receptors could modulate other signaling cascades, including PI3K/AKT, Wnt/β‐catenin, and JAK/STAT, to regulate tumor behavior in HCC [136, 137, 138]. Furthermore, noncanonical notch signaling is essential for the regulation of cell migration and invasion. It upregulates key transcription factors such as Snail and Slug, which inhibit E‐cadherin and promote vimentin and N‐cadherin expression, leading to the loss of cell–cell adhesion, enabling HCC cells to detach and migrate to distant organs [139, 140]. In addition, MMPs are upregulated via notch signaling, facilitating the extracellular matrix degradation and aiding in tumor cell invasion [141].

The contribution of the notch signaling pathway to oncogenesis in HCC has been widely supported by numerous studies. However, some research has also identified tumor‐suppressive effects of this pathway. For instance, an early study have shown that the upregulation of notch1 expression can induce apoptosis in HCC cells by altering the p53/Bcl‐2 balance [142]. In a murine model, activation of notch signaling was found to inhibit HCC [143]. These results may be attributed to different TMEs and liver fibrosis backgrounds [133]. Nevertheless, increasing evidence supports the carcinogenic role of notch in HCC development, and several studies have explored therapeutic strategies targeting this pathway for HCC treatment. Giovannini et al. found that inhibition of notch3 significantly enhanced the apoptotic effects induced by sorafenib in HCC cells [144]. Using a mouse xenograft model, they further demonstrated that notch3 knockout combined with 21 days of sorafenib treatment resulted in a significant antitumor effect in vivo [144]. Additionally, some γ‐secretase inhibitors, such as ginsenoside and PF‐03084014, have also shown antitumor activity against HCC in preclinical and clinical studies [145, 146, 147]. Despite these promising findings, the application of notch pathway inhibitors in HCC is primarily limited to preclinical studies and early‐ Phase clinical trials, with large‐scale clinical trials being scarce. Moreover, due to the bidirectional role of the notch pathway in HCC and its crosstalk with other pathways, notch inhibitors may face challenges related to safety, specificity, and resistance. Therefore, future research may need to focus on combined therapeutic strategies and precision medicine. For example, considering the interplay between the notch signaling and the PD‐1/PD‐L1 axis, Meng et al. found that the combination of a notch inhibitor and an anti‐PD‐1 antibody significantly suppressed tumor growth in breast cancer models [148]. This provides insights for the potential application of notch pathway inhibitors in combination with immune therapies for HCC.

### 2.8 Hh Signaling Pathway

**FIGURE 6:**
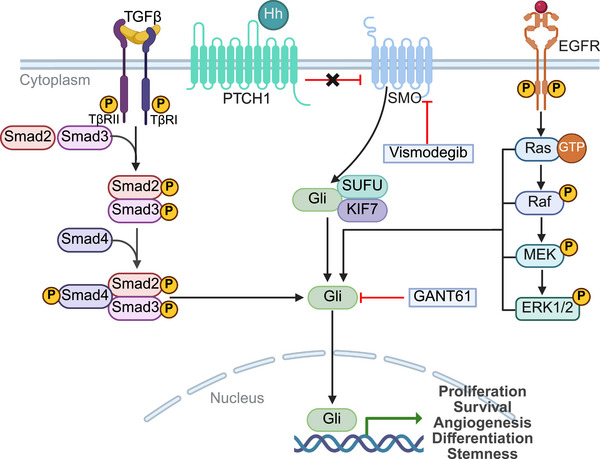
The mechanism of Hh signaling pathway and its crosstalk with other pathways and its related targeted inhibitors. *Abbreviations*: Hh, hedgehog; PTCH1, patched 1; SMO, smoothened; SUFU, suppressor of fused; KIF7, kinesin family member 7; TGF‐β, transforming growth factor beta; TβR, TGF‐β receptor; Smad, signal transducer activator of transcription; EGFR, epidermal growth factor receptor; GTP, guanosine triphosphate; MEK, mitogen‐activated protein kinase kinase; ERK, extracellular signal‐regulated kinase.

The Hh signaling pathway comprises Hh ligands (including Sonic Hh [Shh], Indian Hh [Ihh], and Desert Hh [Dhh]), transmembrane receptors such as Patched (PTCH1/2) and Smoothened (SMO), Gli family transcription factors (Gli1, Gli2, Gli3), and downstream target genes [149]. Under normal conditions, the pathway remains inactive, with PTCH inhibiting SMO [150]. Upon Hh ligand binding to PTCH, the inhibition is relieved, allowing SMO to migrate to the primary cilium and activate the downstream signaling cascade [151]. This activation induces Gli transcription factors to be liberated from their repressor forms, which, in turn, promote the regulation of target gene expression that facilitate cell proliferation, differentiation, and growth (Figure 6) [152].

In most normal liver tissues, the Hh pathway is in a dormant state. However, in HCC, the pathway is aberrantly activated, contributing to tumorigenesis, metastasis, and tumor progression [153]. The activation of Hh signaling in HCC is frequently sustained through autocrine (tumor cell‐intrinsic) and paracrine mechanisms [153, 154], particularly within the TME. For example, Hh ligands secreted by tumor cells can stimulate HSCs, cancer‐associated fibroblasts, and endothelial cells, promoting the formation of a fibrotic niche that supports tumor growth and metastasis [153]. Studies have shown that SMO, PTCH, Shh, and Gli regulate the expression of key genes such as c‐Myc, that contributes to cell cycle progression and tumor cell proliferation [150, 155]. In HCC tissues, inhibition of Gli significantly reduces the expression of Bcl‐2 and c‐Myc, leading to an attenuation of cell cycle progression and inhibition of HCC cell proliferation [156].

Beyond the canonical signaling axis, noncanonical inputs can also activate Gli transcription factors independent of SMO. For instance, TGF‐β/Smad3 and Ras/Raf/MEK/ERK signaling pathways can induce Gli activity, linking the Hh pathway to survival, metabolism, and motility [157, 158]. Moreover, SMO can signal through RhoA, Rac1, and PI3K/AKT/mTOR pathways, promoting tumor cell survival and migration [159, 160].

Components of the Hh pathway have been observed to be significantly overexpressed in HCC. Specifically, nearly 60% of HCC patients exhibit overexpression of Shh, and over 50% show overexpression of PTCH1 and Gli1 [161, 162]. Moreover, aberrant activation of this pathway is strongly correlated with poorer prognosis, metastasis, and chemotherapy resistance [154, 163]. Therefore, targeting these pathway components holds great potential for the treatment of HCC.

Vismodegib is an approved SMO inhibitor used as a therapeutic strategy for advanced basal cell carcinoma [164]. In animal models, Philips et al. found that vismodegib reduced tumor burden and inhibited HCC progression in fibrotic livers [165]. Pinter et al. demonstrated that vismodegib downregulated tumor VEGF production in vitro and reduced VEGF expression, angiogenesis, and tumor growth in an orthotopic rat model [166]. Abou‐Alfa et al. investigated the pharmacokinetics (PK) and safety of vismodegib in patients with varying degrees of liver dysfunction, including those with HCC [167]. They found that liver dysfunction did not appear to affect the PK of vismodegib [167]. However, four patients experienced dose‐limiting toxicities, including one from the moderate liver damage group and three from the severe liver damage group, all of which exhibited hyperbilirubinemia [167]. Despite a significant proportion of individuals with advanced HCC presenting with severe liver cirrhosis, making it difficult to establish a causal relationship between vismodegib exposure and severe AEs, the findings suggest that liver‐related AEs should be closely monitored when using vismodegib to treat HCC. Across in vitro and in vivo models, the GLI direct inhibitor GANT61 significantly inhibited the proliferation and migration abilities of undifferentiated or cancer stem‐like HCC cells, while downregulating stemness/EMT‐related genes such as OCT4, BMI1, CD44, ALDH, and Snail1 [168]. Furthermore, Wang et al. found that GANT61 significantly inhibited the Hh signaling pathway, thereby reversing sorafenib resistance in CD44‐positive HCC cells [163]. These studies highlight the potential of Hh pathway inhibitors in HCC management. However, it merits attention that the Hh pathway intersects with multiple other signaling pathways, and it may have heterogeneous effects depending on the cell type. Therefore, target escape, resistance, or unexpected AEs may occur during treatment. Additionally, as HCC typically occurs in the context of cirrhosis or end‐stage liver disease, with impaired liver function, safety considerations are critical when targeting the Hh pathway for HCC treatment.

## 3 Targeted Therapies in HCC

The HCC treatment landscape has changed substantially as targeted therapies have emerged. These therapies focus on specific molecular drivers of HCC, including dysregulated angiogenesis, cell survival pathways, and oncogenic signaling networks. Unlike traditional chemotherapy, which indiscriminately affects both cancerous and healthy cells, targeted agents are designed to block dysregulated signaling cascades that promote cancer progression and dissemination, thereby reducing off‐target effects and improving treatment efficacy. After years of development, a range of small‐molecule inhibitors and monoclonal antibodies have progressed from development to authorization and are currently used in practice, providing new options for individuals with HCC that is inoperable or at an advanced stage.

### 3.1 First‐Line Targeted Therapies

#### 3.1.1 Sorafenib

Sorafenib, a broad‐spectrum kinase blocker, became the earliest US FDA‐authorized targeted agent for HCC in 2007 [169]. It represents the shift from traditional chemotherapy to molecularly targeted therapies and has since been incorporated into first‐line systemic management for unresectable or metastatic HCC.

Sorafenib primarily exerts its effects by suppressing tumor vascularization and inhibiting cellular growth. Specifically, it inhibits the taget VEGFR‐2, VEGFR‐3, PDGFR‐β, Raf, and c‐Kit to suppress tumor vascularization and inhibit tumor growth [170]. Numerous clinical trials have investigated the effectiveness of sorafenib for treating HCC. As one of the key studies, the SHARP trial, a randomized Phase III trial, confirmed sorafenib's therapeutic efficacy and safety in patients with advanced HCC [171]. The cohort consisted of 602 patients with advanced HCC, all of whom had not been treated with systemic therapy before enrollment. Of these, 299 patients received sorafenib treatment, while 303 patients received a placebo. In terms of primary outcome measures, sorafenib treatment resulted in a median overall survival (mOS) of 10.7 months, whereas the placebo group had a median of 7.9 months (hazard ratio (HR) = 0.69; 95% confidence interval (CI), 0.55–0.87, *p* < 0.001), with survival rates at 1 year of 44% for sorafenib and 33% for placebo, respectively. Regarding secondary outcomes, the median time to progression (mTTP) for the sorafenib and placebo groups were 5.5 and 2.8 months, respectively (HR = 0.58; 95% CI, 0.45–0.74, *p* < 0.001). Despite a greater occurrence of treatment‐related AEs (TRAEs) in the sorafenib arm, the majority of observed events, including diarrhea, weight loss, and hand–foot reaction, were classified as mild to moderate in severity [171].

Since the SHARP study predominantly enrolled participants from Europe and North America, the ORIENTAL Phase III trial was performed across the Asia‐Pacific region to further investigate sorafenib's therapeutic efficacy and safety in advanced HCC patients [172]. The study was carried out over a period from September 20, 2005 to January 31, 2007, across 23 centers located in the Asia‐Pacific region. The study enrolled 226 untreated patients diagnosed with advanced HCC, who were then randomized to either receive sorafenib (150 patients) or a placebo (76 patients). The final results showed that the mOS for the sorafenib group was 6.5 months, compared with 4.2 months for the placebo group (HR = 0.68; 95% CI, 0.50–0.93, *p* = 0.014). The mTTP was 2.8 months for the sorafenib group and 1.4 months for the placebo group (HR = 0.57; 95% CI, 0.42–0.79, *p* = 0.0005). Safety analysis demonstrated that the overall frequency of AEs aligned closely with the findings from the SHARP study, and the incidence of severe AEs was also comparable between the sorafenib and placebo groups (Table 1) [172].

**TABLE 1:** Clinical trials of monotherapy for the treatment of hepatocellular carcinoma.

Line	Drug	Target	Clinical trial	Comparator	OS (months)	PFS (months)	Grade 3/4 AEs	References	Trial identifier
Targeted therapy									
First	Sorafenib	VEGFR‐2, VEGFR‐3, PDGFR‐β, Raf, c‐Kit	SHARP	Placebo	10.7 vs. 7.9	NA	Diarrhea (8 vs. 2%) Hand–foot skin reaction (8 vs. <1%) Hypertension (2 vs. <1%) Abdominal pain (2 vs. 1%)	[171]	NCT00105443
			ORIENTAL	Placebo	6.5 vs. 4.2	NA	Hand–foot skin reaction (10.7 vs. 0) Diarrhea (6.0 vs. 0) Fatigue (3.4 vs. 1.3%) Rash/desquamation (0.7 vs. 0%) Hypertension (2.0 vs. 0%) Nausea (0.7 vs. 1.3%)	[172]	NCT00492752
	Lenvatinib	VEGFR, PDGFR, c‐kit, FGFR, RET	REFLECT	Sorafenib	13.6 vs. 12.3	7.4 vs. 3.7	Palmar‐plantar erythrodysesthesia (3 vs. 11%) Diarrhea (4 vs. 4%) Hypertension (23 vs. 14%) Decreased appetite (5 vs. 1%) Decreased weight (8 vs. 3%)	[176]	NCT01761266
	Donafenib	VEGFR, PDGFR, Raf	ZGDH3	Sorafenib	12.1 vs. 10.3	3.7 vs. 3.6	Hand–foot skin reactions (6 vs. 12%) Diarrhea (2 vs. 3%) Decreased platelet count (4 vs. 2%) Hypertension (9 vs. 9%) Elevated aspartate aminotransferase(2% vs. 5%)	[180]	NCT02645981
Second	Regorafenib	VEGFR, PDGFR, FGFR, TIE2, KIT, RET, Raf	RESORCE	Placebo	10.6 vs. 7.8	3.1 vs. 1.5	Hand–foot skin reaction (13 vs. 1%) Diarrhea (2 vs. 0%) Fatigue (6 vs. 2%) Hypertension (13 vs. 3%) Anorexia (3 vs. 0%)	[185]	NCT01774344
	Cabozantinib	VEGFR, MET, AXL, RET, c‐kit	CELESTIAL	Placebo	10.2 vs. 8.0	5.2 vs. 1.9	Palmar‐plantar erythrodysesthesia (17 vs. 0%) Hypertension (16 vs. 2%) Increased aspartate aminotransferase level (12 vs. 7%) Fatigue (10 vs. 4%) Diarrhea (10 vs. 2%)	[186]	NCT01908426
	Apatinib	VEGFR‐2	AHELP	Placebo	8.7 vs. 6.8	4.5 vs. 1.9	Hypertension (28 vs. 2%) Hand–foot syndrome (18 vs. 0%) Thrombocytopenia (13 vs. 1%)	[192]	NCT02329860
	Ramucirumab	VEGFR‐2	REACH	Placebo	9.2 vs. 7.6	2.8 vs. 2.1	Ascites (5 vs. 4%) Hypertension (12 vs. 4%) Asthenia (5 vs. 2%)	[194]	NCT01140347
			REACH‐2	Placebo	8.5 vs. 7.3	2.8 vs. 1.6	Hypertension (13 vs. 5%) Hyponatremia (6 vs. 0%) Increased aspartate aminotransferase (3 vs. 5%)	[195]	NCT02435433
ICIs									
First	Tislelizuma	PD‐1	RATIONALE‐208	NA	13.2	2.7	Liver transaminase elevations (4%) Increased blood bilirubin (1%)	[203]	NCT03419897
			RATIONALE‐301	Sorafenib	15.9 vs. 14.1	2.1 vs. 3.4	22.2 vs. 53.4%	[204]	NCT03412773
Second	Nivolumab	PD‐1	CheckMate 040	NA	15.0	3.4	25.0%	[200]	NCT01658878
			CheckMate 459	Sorafenib	16.4 vs. 14.7	3.7 vs. 3.8	Palmar‐plantar erythrodysesthesia (<1 vs. 14%) Aspartate aminotransferase increase (6 vs. 4%) Hypertension (0 vs. 7%)	[201]	NCT02576509
	Camrelizumab	PD‐1	NA	NA	13.8	2.1	Increased aspartate aminotransferase (5%) Decreased neutrophil count (3%) Increased blood bilirubin (2%)	[202]	NCT02989922
	Pembrolizuma	PD‐1	KEYNOTE‐224	NA	12·9	4·9	26%	[205]	NCT02702414
			KEYNOTE‐240	Placebo	13.9 vs. 10.6	3.0 vs. 2.8	52.7 vs. 46.3%	[206]	NCT02702401
			KEYNOTE‐394	Placebo	14.6 vs. 13.0	2.6 vs. 2.3	13.3 vs. 5.9%	[207]	NCT03062358

*Abbreviations*: AEs, adverse events; FGFR, fibroblast growth factor receptor; ICIs, immune checkpoint inhibitors; NA, not available; OS, overall survival; PD‐1, programmed cell death protein 1; PDGFR, platelet‐derived growth factor receptor; PFS, progression‐free survival; VEGFR, vascular endothelial growth factor receptor.

#### 3.1.2 Lenvatinib

Sorafenib's approval in 2007 for advanced HCC as a first‐line therapy paved the way for several antiangiogenic agents, such as brivanib [173], sunitinib [174], and linifanib [175], to undergo large‐scale Phase III clinical trials internationally. However, none of them demonstrated efficacy superior to sorafenib in controlling HCC.

In 2018, the US FDA granted approval for lenvatinib as a treatment option for patients with HCC who are not candidates for surgery, offering an alternative to sorafenib. The approval was granted following the findings from the REFLECT trial [176]. Results from the REFLECT trial indicated that lenvatinib was noninferior to sorafenib with respect to OS, while demonstrating superior efficacy compared with sorafenib in progression‐free survival (PFS) and TTP. The experimental group showed a median OS of 13.6 months, while the control group had 12.3 months (HR = 0.92; 95% CI, 0.79–1.06). In terms of PFS, the median was 7.4 months for the treatment group compared with 3.7 months for the control group (HR = 0.66; 95% CI, 0.57–0.77, *p* < 0.0001). The median TTP was 8.9 versus 3.7 months (HR = 0.63; *p* < 0.0001), and the objective response rate (ORR) was 24.1% for the experimental group versus 9.2% for the control group. The occurrence of treatment‐emergent AEs (TEAEs) of Grade ≥3 was comparable between the two groups, with rates of 3.2 and 3.3 patients‐year, respectively. The most common TEAEs in the lenvatinib cohort included hypertension, diarrhea, anorexia, and weight loss, while the most common TEAEs in the sorafenib cohort were hand–foot skin reaction, diarrhea, hypertension, and anorexia. The incidence of treatment‐related fatal AEs was 2 versus 1% for the two groups.

Since lenvatinib was authorized for use in HCC, its potential benefits when combined with other treatment regimens have gradually become a subject of research. The Phase III, randomized LAUNCH trial, led by Peng et al., aimed to examine the efficacy of lenvatinib in combination with transarterial chemoembolization (TACE) for treating advanced HCC as a first‐line approach [177]. The result showed that the lenvatinib‐TACE combination group had a significantly longer median OS (17.8 months) than the lenvatinib group (11.5 months) (HR = 0.45; *p* < 0.001) [177]. Additionally, the median PFS for the lenvatinib‐TACE group was 10.6 months, while the lenvatinib group had a median of 6.4 months (HR = 0.43; *p* < 0.001) [177]. The lenvatinib‐TACE combination group demonstrated a higher ORR compared with the lenvatinib group (54.1 vs. 25.0%, *p* < 0.001) [177].

Lenvatinib shares similarities with sorafenib, both being multitargeted tyrosine kinase blockades that act on common receptors including VEGFR, PDGFR, and c‐kit [178]. The unique mechanism of lenvatini lies in its inhibition of additional key receptors, including FGFR and RET [178]. The combination of lenvatinib and TACE may enhance tumor control through a synergistic mechanism. TACE provides immediate local tumor control by blocking blood supply, while lenvatinib further inhibits angiogenesis and tumor growth, potentially preventing tumor recurrence and improving overall treatment outcomes.

#### 3.1.3 Donafenib

As an oral small molecule, donafenib targets multiple receptor kinases. It exerts its effects by inhibiting VEGFR, PDGFR, and the Ras/Raf/MEK/ERK pathways, thus dual‐blocking tumor cell proliferation and angiogenesis, while also optimizing the immune microenvironment [179]. Its deuterated structure is optimized based on sorafenib, improving its metabolic profile, reducing toxicity, and enhancing efficacy.

A randomized Phase II–III study conducted by Qin et al. assessed the therapeutic effectiveness of donafenib compared with sorafenib as a first‐line management for unresectable or metastatic HCC. from March 2016 to April 2018 [180]. In this study, 668 patients were assigned to the donafenib or sorafenib treatment groups according to an intention‐to‐treat (ITT) analysis, with 328 and 331 patients, respectively, forming the full analysis set. Patients in the donafenib group had a median OS of 12.1 months, significantly longer than the 10.3 months observed in the sorafenib group (HR = 0.831; 95% CI, 0.699–0.988; *p* = 0.0245). This result was also observed in the intention‐to‐treat (ITT) population, where donafenib showed superior OS outcomes compared with sorafenib. The donafenib group had a median PFS of 3.7 months, while the sorafenib group had a median of 3.6 months (*p* = 0.0570). The ORR for donafenib was 4.6%, higher than the 2.7% observed with sorafenib (*p* = 0.2448). In the case of disease control rate (DCR), donafenib achieved 30.8%, whereas sorafenib's DCR was 28.7% (*p* = 0.5532). Notably, donafenib resulted in a significantly smaller proportion of Grade ≥3 TRAEs than sorafenib (38 vs. 50%; *p* = 0.0018). The study indicates that, despite the absence of statistical differences in secondary endpoints, donafenib demonstrated a more significant superiority over sorafenib in OS. Based on this study, in June 2021, donafenib was authorized by the National Medical Products Administration (NMPA) of China for treating unresectable HCC in patients who had not received prior systemic treatment.

The advent of targeted therapies has offered new prospects for individuals with advanced HCC; however, resistance to first‐line targeted therapies remains a significant challenge that requires further investigation. Tumor cells often activate compensatory pathways that contribute significantly to resistance. For instance, the PI3K/AKT/mTOR and Hippo–YAP pathways have been shown to be strongly activated in resistant HCC cells [181, 182]. Additionally, dysregulation of regulated cell death is another cause of resistance. For example, sorafenib induces tumor cell death in HCC by triggering ferroptosis. However, during sorafenib treatment, secernin‐1 acts as a bridge that brings the phosphokinase serine/threonine kinase 38 to the antiferroptosis protein glutathione peroxidase 4, where it phosphorylates glutathione peroxidase 4 to prevent its degradation, thereby reducing lipid peroxidation and inhibiting ferroptosis [183]. In summary, the mechanisms of resistance to first‐line targeted therapies are complicated. In addition to the mechanisms mentioned above, activated cancer stem cell traits, activation of EMT, alterations in TME, and changes in the expression of lncRNAs, circular RNAs, and microRNAs all contribute to the resistance process. Therefore, the development of second‐line therapies has become increasingly important for patients who experience disease progression or intolerable AEs. Second‐line targeted therapies are designed to provide continued treatment options for these patients, often by targeting different molecular mechanisms associated with the advancement of HCC. Drugs such as regorafenib, cabozantinib, and ramucirumab have emerged as key players in the second‐line treatment landscape, offering improved survival outcomes and extended disease control. These therapies are typically employed after the failure of first‐line treatments, providing additional hope for patients with advanced, unresectable, or metastatic HCC.

### 3.2 ‐Line Targeted Therapies

#### 3.2.1 Regorafenib

Regorafenib, an oral small molecule targeting multiple kinases, inhibits critical pathways that drive tumor angiogenesis (VEGFR‐1, ‐2, ‐3, TIE2), tumorigenesis (KIT, RET, RAF‐1, BRAF), and tumor metastasis (VEGFR‐3, PDGFR, FGFR). In a multicenter Phase II study aiming to explore the efficacy of regorafenib as second‐line therapy for intermediate or advanced HCC [184], Bruix et al. reported that regorafenib showed a notable improvement in both OS and PFS. The median OS was 10.6 months, while the median PFS was 3.1 months. The ORR was 10.6%, and the DCR was 64.7%, showing that regorafenib effectively controlled tumor progression in a substantial proportion of patients. In terms of safety, regorafenib was generally well‐tolerated, with the most frequent Grade ≥3 AEs being hypertension, fatigue, hand–foot skin reaction, and diarrhea. In light of the study's results [184], a Phase III randomized trial (RESORCE) was carried out to evaluate the safety and efficacy of regorafenib in individuals with HCC who showed progression during treatment with sorafenib [185]. From May 14, 2013 to December 31, 2015, a total of 573 participants from 152 centers across 21 countries in the Americas, Europe, Australia, and Asia were randomly assigned in a 2:1 ratio to receive regorafenib along with best supportive care (BSC) (*N* = 379) or placebo in combination with BSC (*N* = 194) [185]. The regorafenib group had a median OS of 10.6 months, while the control group had a median of 7.8 months. Regarding secondary endpoints, the regorafenib group demonstrated a median PFS of 3.1 months, significantly longer than the 1.5 months observed in the control group. The regorafenib group had a median TTP of 3.2 months, while the control group showed a median of 1.5 months. The DCR was 65.2% in the regorafenib arm, whereas the control group had a DCR of 36.1%, and the ORR was 10.6 and 4.1%, respectively. Subgroup analyses revealed that regorafenib provided benefits across nearly all subgroups, including age, gender, region, ECOG score, AFP levels, Child‐Pugh score, presence of extrahepatic metastasis, and major vascular invasion, with particularly significant improvements in PFS and TTP. Regarding safety and tolerability, the most commonly observed Grade ≥3 adverse events in the regorafenib arm, such as hypertension, hand–foot skin reaction, fatigue, and diarrhea, were consistent with the drug's known safety profile, in comparison with the control group. Subsequently, in 2017, the US FDA authorized regorafenib as the first approved second‐line treatment for advanced HCC after the failure of sorafenib.

#### 3.2.2 Cabozantinib

As an oral multitarget kinase inhibitor, cabozantinib primarily targets VEGFR, MET, AXL, RET, and KIT, which are involved in promoting tumor blood vessel formation, cancer cell proliferation, survival, and metastasis.

In 2019, cabozantinib received US FDA approval as a second‐line therapy for advanced HCC following sorafenib failure. The CELESTIAL trial [186], a Phase III randomized study, provided the primary evidence for approval, evaluating cabozantinib's effectiveness compared with placebo in patients with advanced HCC who had received previous sorafenib therapy. The study showed that cabozantinib significantly enhanced OS relative to placebo, with the median OS reaching 10.2 months in the treatment group and 8.0 months in the placebo group (*p* = 0.005). Cabozantinib demonstrated a median PFS of 5.2 months, significantly longer than the 1.9 months observed with placebo (*p* < 0.001). The ORR was 4% for the cabozantinib group, compared with under 1% for the placebo group (*p* = 0.009). High‐grade AEs occurred at a rate approximately twice as high in the cabozantinib group compared with the placebo group (68 vs. 36%). Hand–foot skin reaction (17 vs. 0), hypertension (16 vs. 2%), elevated aspartate aminotransferase (AST) (12 vs. 7%), fatigue (10 vs. 4%), and diarrhea (10 vs. 2%) were the most prevalent Grade 3/4 AEs in the cabozantinib and placebo groups [186].

Inspired by the notable enhancement in OS and PFS observed with the combination of cabozantinib and immunotherapy compared with sunitinib as first‐line treatment for renal cell carcinoma [187, 188], the combination of cabozantinib and immunotherapy has been investigated as a potential first‐line treatment option for advanced HCC. Kelley et al. compared cabozantinib combined with atezolizumab to sorafenib for treating advanced‐stage HCC as a first‐line approach (COSMIC‐312) [189]. In the study, the combination therapy group had a median PFS of 6.8 months (99% CI, 5.60–8.3), while the sorafenib group exhibited a median PFS of 4.2 months (99% CI, 2.8–7.0) (HR = 0.63; 99% CI, 0.44–0.91, *p* = 0.0012) [189]. However, no substantial difference in OS was observed between the two groups. The combination therapy group (interim analysis) demonstrated a median OS of 15.4 months (96% CI, 13.7–17.7), while the sorafenib group had a median OS of 15.5 months (12.1 months, not estimable) (HR = 0.90; 96% CI, 0.69–1.18; *p* = 0.44) [189]. In further subgroup analyses, it was found that in the HBV subgroup (*n* = 191), the median OS for the cabozantinib plus atezolizumab group was 18.2 months, compared with 14.9 months in the sorafenib monotherapy group (HR = 0.53; 95% CI, 0.33–0.87). In the HCV subgroup (*n* = 203), the median OS for both groups was similar, with 13.6 months for the combination group and 14.0 months for the sorafenib group (HR = 1.10; 95% CI, 0.72–1.68). For the nonviral subgroup (*n* = 255), the combination therapy arm showed a median OS of 15.2 months, while the sorafenib group did not achieve a median OS (HR = 1.18; 95% CI, 0.78–1.79). These results further validated that HBV‐associated HCC may experience greater therapeutic benefit from cabozantinib plus atezolizumab combination therapy. The potential underlying mechanism might consist in the presentation of immune dysregulation in the disease progression of HBV‐associated HCC. Overexpression of immune inhibitory factors such as PD‐1 and CTLA‐4 leads to T‐cell dysfunction, which contributes to the chronicity of viral infection, the development of self‐limiting hepatitis, and the initiation and progression of HCC. In 2024, the research team reported the final result of COSMIC‐312 trial [190]. It turned out that the combination of cabozantinib and atezolizumab in the first‐line setting did not improve OS versus sorafenib in advanced HCC patients and the PFS benefit of the combination versus sorafenib was maintained [190]. It is reasonable to expect that the addition of a treatment regimen would significantly improve PFS in patients with combining targeted therapy and immunotherapy. Nevertheless, the combination treatment showed no significant benefit in OS and was associated with a greater occurrence of AEs. This raises the consideration that perhaps some subgroups of HCC patients may be more appropriate for monotherapy, while others may benefit from combination therapy. For example, recently, Nagashima et al. reported a case of an advanced HCC patient who progressed after multiple treatment regimens, including sorafenib, lenvatinib, regorafenib, ramucirumab, and combination therapy with atezolizumab and bevacizumab, but achieved complete remission with cabozantinib monotherapy as the sixth‐line treatment [191].

#### 3.2.3 Apatinib

Apatinib is an orally administered TKI that selectively binds to the intracellular adenosine triphosphate binding site of VEGFR‐2, blocking its phosphorylation. This inhibition suppresses signaling pathways such as Ras/Raf/MEK/ERK, and PI3K/AKT/mTOR, which are implicated in the proliferation, migration, and angiogenesis.

The AHELP trial is Phase III randomized study designed to evaluate the clinical outcomes and safety of apatinib in patients with advanced HCC who were ineffective or intolerant to sorafenib or systemic chemotherapy [192]. The findings indicated that the median OS for the apatinib group was 8.7 months (95% CI, 7.5–9.8), while the placebo group had a median OS of 6.8 months (95% CI, 5.7–9.1) (*p* = 0.048). The 6‐month OS rates were 70 and 56.1%, and the 12‐month OS rates were 36.8 and 28.9%, respectively. The apatinib group showed a median PFS of 4.5 months (95% CI, 3.9–4.7), significantly longer than the 1.9 months (95% CI, 1.9–2.0) observed in the placebo group, with a HR of 0.471 (95% CI, 0.369–0.601, *p* < 0.0001). The ORR was 10.7% in the apatinib group and 1.5% in the placebo group. Common Grade 3/4 TRAEs included hypertension (28 vs. 2%), hand–foot syndrome (18 vs. 0), and thrombocytopenia (13 vs. 1%) [192]. These results demonstrate that apatinib significantly improves OS, PFS, and ORR in advanced HCC patients who exhibit resistance to or intolerance of previous systemic treatments, with manageable safety (Table 1). The AHELP study led to the approval of apatinib by the NMPA on December 31, 2020, for its application in the second‐line management of advanced HCC.

#### 3.2.4 Ramucirumab

Ramucirumab is an IgG1 monoclonal antibody designed to specifically targets VEGFR‐2, inhibiting the activation of signaling pathways that promote the angiogenesis within tumors.

In a Phase II study, ramucirumab has demonstrated preliminary antitumor activity, accompanied by tolerable toxicity in advanced HCC [193]. Zhu et al. evaluated the effectiveness and safety of ramucirumab as a second‐line option in advanced HCC after sorafenib therapy (REACH). There was no significant improvement in OS with ramucirumab compared with placebo, as the median OS was 9.2 months (95% CI, 8.0–10.6) for the treatment group, while the placebo group had a median OS of 7.6 months (95% CI, 6.0–9.3) (HR = 0.87; 95% CI, 0.72–1.05; *p* = 0.14) [194]. However, in the subgroup analysis, it was found that individuals with a baseline AFP level of 400 ng/mL or greater were more likely to benefit from ramucirumab, with the treatment group having a median OS of 7.8 months (95% CI, 5.8–9.3), while the placebo group showed a median of 4.2 months (95% CI, 3.7–4.8) [194]. Subsequently, the research team conducted a Phase III trial to assess the therapeutic potential of ramucirumab in advanced HCC patients with baseline AFP ≥400 ng/mL (REACH‐2) and the outcomes were promising [195]. The median OS (8.5 vs. 7.3 months; *p* = 0.0199) and PFS (2.8 vs. 1.6 months; *p* < 0.0001) in the ramucirumab group were significantly improved compared with the placebo group [195], which rendered the study the first Phase III trial to demonstrate a positive result in a biomarker‐selected HCC population. In 2019, ramucirumab received US FDA approval for the treatment of advanced HCC in patients with an AFP level of 400 ng/mL or higher.

The potential mechanisms underlying the efficacy of ramucirumab in HCC patients with baseline AFP ≥400 ng/mL may be as follows: high AFP levels often correspond to rapidly proliferating or large tumors that readily develop hypoxic regions, leading to the stability of HIF‐1α and transcriptional upregulation of VEGF‐A and multiple proangiogenic genes [196, 197], while concurrently promoting activation of endothelial VEGFR‐2 signaling. When the angiogenic burden is higher, VEGFR‐2 blockade is more likely to produce meaningful microenvironmental improvements (enhanced perfusion, reduced interstitial pressure, and alleviated hypoxia), which can translate clinically into PFS/OS benefits. Moreover, existing studies have demonstrated that AFP can activate the PI3K/AKT pathways, thereby enhancing angiogenesis and proliferation in HCC [198, 199]. This may potentially involve a positive feedback loop with the HIF‐1α/VEGF axis induced by hypoxia. These findings provide strong support for understanding the potential association between high AFP levels and a proangiogenic state. Apatinib is also a VEGFR‐2 inhibitor. Findings from the CARES‐310 study indicate that it confers a marked advantage in HCC with AFP >400. Unlike ramucirumab, however, apatinib also demonstrated an improvement in survival outcomes for patients with AFP <400, which may reflect the contribution of combination therapy with other immunotherapeutic agents.

## 4 Immunotherapies in HCC

Liver tissue harbors abundant immunosuppressive cell populations and exhibits intrinsic immune tolerance, thereby preventing potential damage from autoimmunity. Cancer cells, however, exploit this tolerogenic milieu to avoid immune surveillance. By disrupting inhibitory signaling between tumor and immune cells, ICIs can reverse such escape, reactivating immune effectors to eliminate malignant cells. Monoclonal antibodies targeting PD‐1/PD‐L1 and CTLA‐4 inhibitors are among the most commonly applied ICIs. In HCC, single‐agent immunotherapy is generally employed as second‐line therapy, whereas first‐line treatment primarily consists of combination regimens, including targeted therapy plus immunotherapy and dual‐ICI combinations.

### 4.1 Monoimmunotherapy

#### 4.1.1 Nivolumab and Camrelizumab

Nivolumab is a human monoclonal antibody designed to block the binding of PD‐1 to PD‐L1. In the CheckMate 040 trial, the efficacy and safety of nivolumab were investigated in patients with advanced HCC [200]. According to the results, nivolumab treatment offers clinical benefits and a favorable safety profile in individuals with advanced HCC, independent of previous sorafenib use. Following the findings of the CheckMate 040 trial, the US FDA granted nivolumab approval for second‐line treatment in HCC patients. The efficacy of nivolumab for first‐line treatment of HCC has also been investigated. In the CheckMate 459 study, Yau et al. assessed nivolumab monotherapy compared with sorafenib monotherapy in the first‐line treatment for patients with advanced HCC [201]. However, it turned out that nivolumab did not lead to a significant increase in OS compared with sorafenib [201].

Camrelizumab is also a humanized anti‐PD‐1 monoclonal antibody. In a multicenter, Phase II trial, Qin et al. explored the therapeutic potential of camrelizumab in patients with advanced HCC who had been treated previously [202]. Camrelizumab demonstrated antitumor efficacy in patients with HCC who experienced progression or intolerance to prior systemic treatments, with an ORR of 14.7% (95% CI, 10.3–20.2%) and a 6‐month OS probability of 74.4% (95% CI, 68.0–79.7%) [202]. This research contributed to the NMPA's approval of camrelizumab for treating HCC in the second‐line setting.

#### 4.1.2 Tislelizumab

Tislelizumab is an IgG4 humanized antibody designed to inhibit PD‐1. As a PD‐1 monoclonal antibody with modifications to the Fc region, it lowers the affinity for Fcγ receptors on macrophages, which in turn disrupts the phagocytic action facilitated by antibodies. This modification prevents the decrease of effector T cells from affecting the antitumor efficacy, while boosting the immune system's capacity to recognize and eliminate tumor cells.

The RATIONALE‐208 study was an international Phase II trial evaluating the clinical outcomes and safety of single‐agent tislelizumab in patients with previously treated advanced HCC [203]. According to the study, tislelizumab showed sustained objective responses in patients with advanced HCC who had undergone previous therapies, regardless of prior treatment lines, and exhibited a manageable safety profile [203]. On the basis of this study, tislelizumab received formal regulatory approval from China's NMPA for use as a treatment option after first‐line therapies in patients with unresectable HCC.

The RATIONALE‐301 study by Qin et al. evaluated tislelizumab as a first‐line treatment for patients with unresectable HCC, focusing on its efficacy and safety [204]. The Phase III randomized clinical trial revealed that the median OS of patients treated with tislelizumab was 15.9 (95% CI, 13.2–19.7) months, compared with 14.1 (95% CI, 12.6–17.4) months in patients treated with sorafenib, indicating an OS noninferiority of tislelizumab versus sorafenib [204]. The occurrence of TRAEs was reported in 96.2% (325 out of 338) of patients treated with tislelizumab and 100% (324 out of 324) of patients treated with sorafenib [204]. Grade 3 or higher TRAEs were observed in 22.2% (75 patients) of those receiving tislelizumab and 53.4% (173 patients) of those receiving sorafenib. Subsequently, in January 2024, the NMPA of China approved tislelizumab as a monotherapy for first‐line management of unresectable or metastatic HCC.

#### 4.1.3 Pembrolizumab

Pembrolizumab is an engineered monoclonal antibody that specifically inhibits PD‐1. According to the KEYNOTE‐224 trial, pembrolizumab exhibited significant efficacy and acceptable tolerability in patients with HCC who are either intolerant to sorafenib or have experienced disease progression following sorafenib treatment, resulting in an ORR of 17% (95% CI, 11–26) [205]. Thereafter, the US FDA granted pembrolizumab an indication for HCC in the second‐line setting.

In the KEYNOTE‐240 study, the analysis did not meet the protocol‐defined criteria for statistical significance for OS or PFS; however, the median OS and PFS were longer with pembrolizumab plus BSC than with placebo plus BSC [206]. The KEYNOTE‐394 trial evaluated the efficacy of pembrolizumab plus BSC versus placebo plus BSC in patients from Asia with previously treated advanced HCC [207]. The pembrolizumab arm yielded an median OS of 14.6 months, whereas the placebo arm had a median of 13.0 months; the HR for death was 0.79 (95% CI, 0.63–0.99), with *p* = 0.0180 [207]. Additionally, the pembrolizumab group demonstrated a longer median PFS (2.6 vs. 2.3 months; HR for progression or death = 0.74; 95% CI, 0.60–0.92; *p* = 0.0032) and a higher ORR (12.7 vs. 1.3%; *p* < 0.0001) than the placebo group. Collectively, these studies corroborate global evidence that pembrolizumab confers clinical benefit in advanced HCC following progression on prior systemic therapy and further substantiate its role as a single‐agent option in the second‐line setting (Table 1).

### 4.2 Dual Immunotherapy

#### 4.2.1 Tremelimumab plus Durvalumab

Tremelimumab is a fully humanized IgG2 anti‐CTLA‐4 monoclonal antibody and durvalumab is a human anti‐PD‐L1 antibody. In the HIMALAYA study, three distinct regimens were assessed for their clinical benefit as first‐line treatment options in HCC: durvalumab monotherapy, single tremelimumab regular interval durvalumab (STRIDE), and sorafenib [5]. The results showed that the median OS in the STRIDE group was 16.43 months, compared with 13.77 months in the sorafenib group (HR = 0.78; *p* = 0.0035). Additionally, durvalumab monotherapy demonstrated noninferior median OS compared with sorafenib (HR = 0.86, noninferiority margin 1.08) [5]. In light of the HIMALAYA findings, the STRIDE regimen subsequently received US FDA authorization for use as a first‐line therapy in advanced HCC.

Recently, Rimassa et al. reported the 5‐year OS from the HIMALAYA study [208]. The STRIDE regimen group demonstrated a 5‐year OS rate 2.09 times higher than the sorafenib group (19.6 vs. 9.4%), with a 24% reduction in the risk of death (HR = 0.76; 95% CI, 0.65–0.89; *p* = 0.0008) [208]. Among patients who achieved disease control, the 5‐year OS rate in the STRIDE group was 28.7%, which was 2.26 times higher than the sorafenib group (12.7%). In patients with a tumor size reduction greater than 25%, the 5‐year OS rate in the STRIDE group was 50.7%, compared with 26.3% in the sorafenib group. These promising results further reinforce the position of STRIDE regimen as a cornerstone in first‐line treatment for HCC (Table 2).

**TABLE 2:** Clinical trials of combination therapy for the treatment of hepatocellular carcinoma.

Combined therapy	Drugs (and targets)	Clinical trial	Comparators	mOS (months)	mPFS (months)	ORR (%)	Grade 3/4 AEs	References
Dual ICIs	Tremelimumab + durvalumab (CTLA‐4 + PD‐L1)	HIMALAYA	STRIDE, durvalumab monotherapy, and sorafenib	16.43 vs. 16.56 vs. 13.77	3.78 vs. 3.65 vs. 4.07	20.1 vs. 17.0 vs. 5.1	25.8 vs. 12.9 vs. 36.9%	[5]
	Nivolumab + ipilimumab (PD‐1 + CTLA‐4)	CheckMate 9DW	Sorafenib or lenvatinib	23.7 vs. 20.6	9·1 vs. 9.2	36 vs. 13	41 vs. 42%	[209]
Targeted therapy plus ICI	Atezolizumab + bevacizumab (PD‐L1 + VEGF)	IMbrave150	Sorafenib	NE vs. 13.2	6.8 vs. 4.3	27.3 vs. 11.9	56.5 vs. 55.1%	[4]
	Sntilimab + IBI305 (PD‐1 + VEGF)	ORIENT‐32	Sorafenib	NR vs. 10.4	4.6 vs. 2.8	21.0 vs. 4.0	33 vs. 36%	[219]
	Toripalimab + bevacizumab (PD‐1 + VEGF)	HEPATORCH	Sorafenib	20.0 vs. 14.5	5.8 vs. 4.0	25.0 vs. 6.0	63 vs. 61%	[220]
	Finotonlimab + SCT510 (PD‐1 + VEGF)	NA	Sorafenib	22.1 vs. 14.2	7.1 vs. 2.9	32.0 vs. 4.3	52.6 vs. 37.9%	[221]
	Camrelizumab + apatinib (PD‐1 + VEGFR2)	CARES‐310	Sorafenib	22.1 vs. 15.2	5.6 vs. 3.7	25.0 vs. 6.0	81.0 vs. 52.0%	[223]
	Anlotinib + penpulimab (VEGFR1–3, FGFR1–4, PDGFR‐α/β, and c‐kit + PD‐1)	APOLLO	Sorafenib	16.5 vs. 13·2	6.9 vs. 2.8	16.0 vs. 3.0	50.0 vs. 47.0%	[231]
	Lenvatinib + pembrolizumab (VEGFR, PDGFR, c‐kit, FGFR, RET + PD‐1)	LEAP‐002	Lenvatinib + placebo	21.2 vs. 19.0	8·2 vs. 8.0	26·1 vs. 17.5	62.0 vs. 57.0%	[233]
	TACE + lenvatinib + pembrolizumab (VEGFR, PDGFR, c‐kit, FGFR, RET + PD‐1)	LEAP‐012	TACE + dual placebo	NA	14·6 vs. 10.0	47.0 vs. 33.0	70.0 vs. 31.0%	[234]
	TACE + envafolimab + lenvatinib (PD‐L1 + EGFR, PDGFR, c‐kit, FGFR, RET)	CISLD‐12	NA	NA	7.58	50.0	52.6%	[235]
	Regorafenib + nivolumab (VEFGR, PDGFR, FGFR, TIE2, c‐kit, RET, Raf + PD‐1)	RENOBATE	NA	NR	7.38	31.0	23.8%	[236]

*Abbreviations*: AEs, adverse events; CTLA‐4, cytotoxic T‐lymphocyte‐associated protein 4; EGFR, epidermal growth factor receptor; FGFR, fibroblast growth factor receptor; ICIs, immune checkpoint inhibitors; NA, not available; NE, could not be evaluated; NR, not reached; ORR, objective response rate; OS, overall survival; PDGFR, platelet‐derived growth factor receptor; PD‐L1, programmed death‐ligand 1; PFS, progression‐free survival; STRIDE, single tremelimumab regular interval durvalumab; TACE, transarterial chemoembolization; VEGF, vascular endothelial growth factor.

#### 4.2.2 Nivolumab plus Ipilimumab

In the CheckMate 459 trial, first‐line administration of nivolumab did not confer a statistically significant OS benefit relative to sorafenib in patients with HCC. In contrast, the CheckMate 9DW investigation demonstrated that dual immune checkpoint blockade with nivolumab plus ipilimumab—a CTLA‐4 inhibitor—resulted in validated clinical efficacy in the first‐line setting [209]. According to the CheckMate 9DW trial, eligible participants underwent randomized allocation to a treatment strategy consisting of combined nivolumab and ipilimumab therapy or to a control regimen including lenvatinib or sorafenib [209]. The median OS was 23.7 months in the combination immunotherapy cohort and 20.6 months in the control cohort, corresponding to a statistically significant improvement in survival outcomes (HR = 0.79; *p* = 0.018) [209]. In addition, the ORR occurred in 36% of patients in the combination immunotherapy cohort (121/335; 95% CI, 31–42%) versus 13% in the control cohort (44/333; 95% CI, 10–17%), demonstrating a statistically significant difference (*p* < 0.0001). Regarding safety, 137 out of 332 patients (41%) in the combination immunotherapy cohort and 138 out of 325 patients (42%) in the control cohort experienced Grade 3/4 TRAEs. These results indicate that nivolumab combined with ipilimumab significantly prolonged OS compared with lenvatinib or sorafenib in first‐line treatment of HCC, with manageable safety. Subsequently, regulatory approval for nivolumab in combination with ipilimumab as first‐line systemic therapy for HCC was subsequently granted by the NMPA and US FDA.

#### 4.2.3 Cadonilimab and QL1706

Compared with monotherapy, dual immunotherapy indeed demonstrates superior antitumor efficacy and significantly improves prognosis. However, the incidence of irAEs associated with dual immunotherapy is also higher. Therefore, immunotherapeutic agents that target both PD‐1 and CTLA‐4 may potentially address this issue.

Cadonilimab is a dual‐target ICI targeting PD‐1 and CTLA‐4. The Fc region knockout of it was designed to enhance antitumor activity while reducing AEs. In a Phase Ib/II trial investigating the safety and efficacy of cadonilimab for patients with advanced solid tumors including HCC who had at least one previous systemic therapy (COMPASSION‐03), Gao et al. found that Grade 3/4 TRAEs occurred in 67 (28%) of 240 patients, and the ORR in the HCC cohort was 16.7% [210]. Besides, the Phase Ib/II COMPASSION‐08 study led by Qiao et al. investigated cadonilimab combined with lenvatinib in the first‐line treatment for advanced HCC, demonstrating measurable antitumor activity with a favorable safety profile [211]. In a real‐world study, it was found that cadonilimab plus TKI showed favorable safety and efficacy in the treatment of unresectable HCC, especially when used as first‐line systemic therapy [212]. Currently, a Phase II study (NCT06984718) is underway to compare cadonilimab combined with lenvatinib versus lenvatinib monotherapy in patients with advanced HCC who have experienced disease progression after prior treatment with atezolizumab and bevacizumab.

QL1706 is a combination antibody consisting of PD‐1 monoclonal antibody and CTLA‐4 monoclonal antibody, which are mixed in a fixed ratio of approximately 2:1. It has demonstrated antitumor activity across a range of malignancies, including non‐small cell lung cancer, nasopharyngeal carcinoma, and cervical cancer [213, 214, 215]. In a Phase I/Ib study evaluating the efficacy of QL1706 in patients with advanced solid tumors, QL1706 demonstrated limited efficacy in HCC, probably due to the small sample size [215]. The DUBHE‐H‐308 study is a randomized Phase II/III clinical trial exploring the combination of QL1706 with bevacizumab and/or chemotherapy as first‐line treatment for advanced HCC. In 2024, the investigators reported encouraging preliminary efficacy of QL1706 combined with bevacizumab and chemotherapy in the first‐line treatment of advanced HCC, with a DCR of 87.1%, and a controllable safety profile [216]. In the future, drugs with dual capabilities targeting both PD‐1 and CTLA‐4 may become a new standard treatment option for advanced HCC.

### 4.3 Targeted Therapy in Combination With Immunotherapy

#### 4.3.1 Atezolizumab plus Bevacizumab

Bevacizumab is a monoclonal antibody that targets VEGF. A Phase II trial assessing the effects of bevacizumab in unresectable HCC demonstrated that bevacizumab exhibited notable clinical and biological activity in nonmetastatic HCC, with an ORR of 13% (95% CI, 3–23%) and a PFS rate of 65% at 6 months [217].

Atezolizumab is a humanized IgG1 antibody against PD‐L1 that prevents PD‐L1 from engaging its receptors, thereby attenuating inhibitory checkpoint signaling. In IMbrave150, a pivotal randomized Phase III study, Finn et al. revealed that atezolizumab plus bevacizumab has demonstrated significantly better efficacy over sorafenib in unresectable HCC [4]. The OS rate for the combination group was 67.2% at 12 months, while the sorafenib group had a survival rate of 54.6%. Median PFS was 6.8 months with the combination regimen, whereas sorafenib yielded a median of 4.3 months. And the incidences of severe (Grade 3–4) AEs were comparable across the two groups (Table 2) [4]. In 2020, atezolizumab in combination with bevacizumab received regulatory authorization from both US FDA and the NMPA for use as first‐line systemic therapy in unresectable or metastatic HCC.

The IMbrave050 Phase III randomized trial conducted by Qin et al. examined whether postoperative adjuvant atezolizumab combined with bevacizumab improves outcomes compared with active surveillance among individuals with HCC at high risk of recurrence after complete tumor removal or curative local ablation [218]. In postoperative high‐risk recurrence HCC patients, adjuvant therapy consisting of atezolizumab in combination with bevacizumab was associated with longer recurrence‐free survival according to the Independent Review Committee (IRC), relative to active surveillance without anticancer treatment (HR = 0.72 (95% CI, 0.56–0.93); *p* = 0.012). Findings from IMbrave050 extend the clinical rationale for atezolizumab combined with bevacizumab beyond its established role in the systemic management of unresectable HCC, supporting its potential utility as an adjuvant approach after curative‐intent local therapy.

#### 4.3.2 Other Immunotherapy Agents in Combination With Bevacizumab

Following the establishment of atezolizumab plus bevacizumab as a standard first‐line systemic option for HCC, multiple studies have subsequently explored bevacizumab‐based pairings with alternative ICIs.

The ORIENT‐32 trial by Ren et al. investigated whether sintilimab combined with the bevacizumab biosimilar (IBI305) could improve efficacy outcomes compared with sorafenib in Chinese patients with HBV‐related unresectable HCC [219]. In the combination arm (sintilimab with IBI305), median PFS reached 4.6 months (95% CI, 4.1–5.7), whereas sorafenib achieved 2.8 months (95% CI, 2.7–3.2); this translated into a stratified HR of 0.56 (95% CI, 0.46–0.70; *p* < 0.0001) [219]. At the initial interim analysis for OS, patients receiving sintilimab combined with IBI305 showed superior OS relative to sorafenib (median not reached (95% CI, not reached–not reached) vs. 10.4 months (8.5–not reached); HR = 0.57; 95% CI, 0.43–0.75; *p* < 0.0001) [219]. Subsequently, sintilimab plus IBI305 received NMPA authorization for first‐line management of HCC.

The HEPATORCH study was designed to assess toripalimab administered alongside bevacizumab, with sorafenib serving as the control regimen, in patients with advanced HCC for first‐line treatment [220]. In comparison with sorafenib, toripalimab plus bevacizumab significantly prolonged PFS (median PFS: 5.8 months (95% CI, 4.6–7.2) vs. 4.0 months (95% CI, 2.8–4.2); HR = 0.69 (95% CI, 0.53–0.91); *p* = 0.0086) [220]. Additionally, toripalimab combined with bevacizumab significantly improved OS compared with sorafenib (median OS: 20.0 months (95% CI, 15.3–23.4) vs. 14.5 months (95% CI, 11.4–18.8); HR = 0.76 (95% CI, 0.58–0.99); *p* = 0.039) [220]. In terms of safety, Grade 3 or higher AEs occurred in 102 patients in the toripalimab plus bevacizumab group and in 100 patients in the sorafenib group. On the basis of these results, the NMPA endorsed toripalimab administered with bevacizumab as a first‐line therapy for unresectable or metastatic HCC patients.

Zhao et al. reported a randomized Phase II/III study that assessed a regimen consisting of finotonlimab and the bevacizumab biosimilar (SCT510), using sorafenib as the comparator, in HCC not eligible for surgical resection [221]. It turned out that the finotonlimab plus SCT510 group was associated with longer PFS (7.1 months (95% CI, 6.1–8.4)) compared with sorafenib group (2.9 months (95% CI, 2.8–4.1); stratified HR = 0.5; 95% CI, 0.38–0.65, *p* < 0.0001) [221]. The median OS in patients receiving finotonlimab in combination with SCT510 (22.1 months (95% CI, 18.6–not available)) was also significantly longer than that in patients treated with sorafenib (14.2 months (95% CI, 10.2–15.8); HR = 0.60 (95% CI, 0.44–0.81), *p* < 0.0008) [221]. Despite the absence of current regulatory authorization for finotonlimab paired with SCT510 in advanced HCC, available evidence suggests this regimen may represent an alternative candidate for first‐line systemic therapy.

In EMERALD‐1 study, a global trial enrolling 616 patients, the efficacy of adding durvalumab and/or bevacizumab TACE in first‐line treatment of HCC were evaluated [222]. Patients were randomized receive to durvalumab plus bevacizumab with TACE, durvalumab with TACE, or placebo with TACE. Compared with placebo plus TACE, durvalumab plus bevacizumab combined with TACE prolonged median PFS by 6.8 months (15.0 vs. 8.2 months), whereas durvalumab plus TACE extended PFS by 1.8 months (10.0 vs. 8.2 months) [222]. The study demonstrated the potential of durvalumab plus bevacizumab with TACE in the first‐line treatment of HCC. Follow‐up for EMERALD‐1 study is ongoing. The durvalumab plus bevacizumab with TACE regimen might provide a new first‐line therapeutic option for HCC in the future.

#### 4.3.3 Camrelizumab in Combination With Apatinib

In the CARES‐310 study, Qin et al. compared first‐line camrelizumab plus apatinib with sorafenib for unresectable HCC, focusing on clinical benefit and safety [223]. Between June 28, 2019 and March 24, 2021, a total of 543 patients underwent randomization and were allocated to camrelizumab–apatinib group (*n* = 272) or sorafenib group (*n* = 271). In the primary analysis for PFS on May 10, 2021, the median follow‐up was 7.8 months (IQR 4.1–10.6). Camrelizumab–apatinib yielded significantly longer PFS than sorafenib. The PFS estimate was 5.6 months (95% CI, 5.5–6.3) in the camrelizumab–apatinib arm and 3.7 months (95% CI, 2.8–3.7) in the sorafenib arm [223]. At the interim analysis for OS on February 8, 2022, the median follow‐up was 14.5 months (IQR 9.1–18.7). A reduction in the risk of death was observed with camrelizumab–apatinib (HR = 0.62; 95% CI, 0.49–0.80; one‐sided *p* < 0.0001), alongside OS of 22.1 months (95% CI, 19.1–27.2) and 15.2 months (95% CI, 13.0–18.5) for the camrelizumab–apatinib and sorafenib arms, respectively [223]. The most common Grade 3 or 4 TRAEs were hypertension (38 vs. 15%), palmar‐plantar erythrodysesthesia syndrome (12 vs. 15%), increased AST (17 vs. 5%), and increased alanine aminotransferase (13 vs. 3%). Serious TRAEs occurred in 66 (24%) participants receiving camrelizumab–apatinib and in 16 (6%) participants receiving sorafenib. Based on results of CARES‐310 study, camrelizumab plus apatinib received regulatory authorization from the NMPA for first‐line treatment of HCC. Furthermore, after 16 months of extended follow‐up, the final analysis of the study showed that the median OS in the camrelizumab–apatinib group reached 23.8 months, an extension of 8.6 months compared with the sorafenib group (23.8 vs. 15.2 months; HR = 0.64; 95% CI, 0.52–0.79; one‐sided *p* < 0.0001) [224].

To our knowledge, CARES‐310 study represents the earliest Phase III evidence in previously untreated HCC to meet both primary endpoints using an anti‐PD‐1/PD‐L1 agent paired with a small‐molecule TKI. The differing results compared with other studies, such as COSMIC‐312 and LEAP‐002, may be due to the more selective targeting mechanism of apatinib. VEGFR‐3 is a receptor located on endothelial cells, which, upon binding with its ligands VEGF‐C and VEGF‐D, activates multiple signaling pathways that promote the proliferation, migration, branching, and maturation of lymphatic endothelial cells, thus playing a critical role in lymphangiogenesis [225, 226, 227]. In immune responses, lymphatic vessels serve as an important pathway for T cell migration. The migration of T cells to tumor sites facilitates their infiltration and activation against tumor cells. Apatinib, as a single‐target TKI, primarily targets VEGFR‐2 without inhibiting VEGFR‐3, allowing it to suppress tumor angiogenesis without interfering with lymphangiogenesis. This selective inhibition of VEGFR‐2, while avoiding the impact on VEGFR‐3, enables better synergy with ICIs, enhancing the effectiveness of immunotherapy.

#### 4.3.4 Anlotinib in Combination With Penpulimab

Anlotinib is an orally available, multitarget TKI that suppresses tumor angiogenesis and proliferation by inhibiting VEGFR1‐3, FGFR1‐4, PDGFR‐α/β, and c‐Kit. A Phase II study indicated that anlotinib provides clinically meaningful activity with an acceptable tolerability profile when used as first‐ or second‐line treatment for advanced or metastatic HCC [228].

Penpulimab is a humanized IgG1 monoclonal antibody targeting PD‐1 with the Fc region was reconstituted aiming to potentially reduce subsequent immune‐related AEs. Zheng et al. described penpulimab monotherapy as generally well tolerated and associated with encouraging signals of antitumor effect among individuals with advanced upper gastrointestinal cancers, including HCC [229].

In a Phase Ib/II trial (AK105‐203), Han et al. reported first‐line activity of anlotinib–penpulimab in HCC, with an ORR of 31.0% (95% CI, 15.3–50.8) and a DCR of 82.8% (95% CI, 64.2–94.2) [230]. Median PFS/TTP were 8.8 months (95% CI, 4.0–12.3/4.0–12.9), and the 12‐month OS rate was 69.0% (95% CI, 48.9–82.5). Tolerability was manageable, with high‐grade TRAEs (Grade ≥3) occurred in 19.4% of patients (6/31) [230]. Zhou et al. conducted a Phase III study in previously untreated HCC, evaluating anlotinib paired with penpulimab against sorafenib, with both clinical outcomes and tolerability assessed [231]. According to the results, both PFS and OS favored anlotinib plus penpulimab over sorafenib [231]. The IRC‐assessed median PFS was 2.8 months (95% CI, 2.7–4.1) and 6.9 months (95% CI, 5.8–8.0) (HR = 0.52), while the median OS was 13.2 months (95% CI, 9.7–16.9) and 16.5 months (95% CI, 14.7–19.0) (HR = 0.69), respectively [231]. In terms of safety, the incidence of any grade and Grade 3 or worse TEAEs and TRAEs was similar between the two groups. The most common Grade 3 or worse TRAEs were hypertension (17 vs. 10%) and thrombocytopenia (9 vs. 6%) [231].

#### 4.3.5 Lenvatinib in Combination With Pembrolizumab

The KEYNOTE‐524 trial demonstrated the promising antitumor activity of lenvatinib in HCC in combination with pembrolizumab with manageable toxicities [232]. LEAP‐002, a Phase III trial in HCC, evaluated pembrolizumab added to lenvatinib and observed clinical antitumor activity; nonetheless, superiority for OS and PFS was not statistically confirmed under the protocol‐specified criteria when assessed against lenvatinib monotherapy [233]. Kudo et al. conducted the Phase III LEAP‐012 trial to evaluate a regimen integrating TACE with lenvatinib and pembrolizumab for nonmetastatic unresectable HCC [234]. A reduction in progression risk was observed when lenvatinib and pembrolizumab were added to TACE (HR 0.66; 95% CI, 0.51–0.84; one‐sided *p* = 0.0002), with PFS estimates of 14.6 months (95% CI, 12.6–16.7) versus 10.0 months (95% CI, 8.1–12.2) for the active regimen and the placebo control, respectively [234]. Moreover, the PFS in the Chinese subgroup extended to 16.6 months in the TACE combined with lenvatinib plus pembrolizumab group. Although the OS for this study are not yet fully mature, the TACE combined with lenvatinib plus pembrolizumab regimen has demonstrated a favorable trend, with a 24‐month OS rate of 75% (95% CI, 68–80) versus 69% (62–74) in the TACE with placebo group (HR 0.80 (95% CI, 0.57–1.11); one‐sided *p* = 0.087) [234]. On the basis of this study, on June 10, 2025, the NMPA of China granted regulatory approval for TACE combined with lenvatinib and pembrolizumab as a first‐line therapeutic approach in patients with unresectable, nonmetastatic HCC.

#### 4.3.6 Other Targeted Therapy–Immunotherapy Combinations

Building on the complementary mechanisms of targeted agents and immunotherapy and the positive results of multiple Phase III trials, targeted therapy–immunotherapy combinations are emerging as a mainstay for advanced HCC. Nonetheless, many patients experience disease progression or treatment intolerance with currently available regimens. Accordingly, investigators are evaluating alternative targeted‐immunotherapy combinations for efficacy in advanced HCC.

Chen et al. conducted a Phase II study evaluating a regimen consisting of TACE together with envafolimab and lenvatinib for patients with unresectable HCC. An ORR of 50% and a DCR of 83.3% were observed, with a median PFS of 7.58 months [235]. However, the sample size in this efficacy analysis was small, with only 36 patients included. Further prospective evaluation in larger, well‐designed studies is warranted to validate the therapeutic benefit observed with this regimen.

RENOBATE, a noncomparative Phase II trial from South Korea, evaluated regorafenib plus nivolumab in previously untreated HCC [236]. The trial included 42 participants, and the ORR was 31.0% in the ITT analysis. Among them, one patient (2.4%) achieved complete remission, and 12 patients (28.6%) achieved partial remission [236]. After a median follow‐up of 11.1 months, median PFS was 7.38 months (95% CI, 4.12–13.0), and the 12‐month PFS estimate was 37.8% [236]. At the time of analysis, median OS remained unreached, while OS rate at 12 months was 80.5% [236].

## 5 Conclusion

Recent years have witnessed a marked shift in HCC management, with clinical therapeutic benefits increasingly attributable to targeted therapies and immunotherapies. These approaches have transformed the therapeutic landscape, offering new avenues for managing this challenging disease. Targeted therapies, including small molecule inhibitors and monoclonal antibodies, have shown promising results in blocking key signaling pathways involved in tumor growth, angiogenesis, and metastasis. ICIs have demonstrated encouraging efficacy by reinvigorating the immune system's ability to recognize and eliminate tumor cells. However, despite these advancements, challenges remain. Many patients still exhibit limited sensitivity to treatment, and AEs associated with these therapies continue to be a major concern. Therefore, priority should be given to establishing robust, clinically actionable biomarkers to enable treatment individualization. This would assist clinicians in tailoring therapies to individual patients, maximizing efficacy while minimizing unnecessary AEs.

One of the key challenges in HCC treatment is the lack of sufficient tumor characterization prior to systemic therapy. HCC is one of the few malignancies that can be clinically diagnosed without the need for biopsy, which limits our understanding of the tumor's intrinsic characteristics and hinders the optimization of treatment strategies. In the future, a greater reliance on biopsy may emerge in advanced HCC, particularly for understanding the molecular profile of the tumor. This, coupled with the growing potential of liquid biopsy technologies, may provide complementary approaches to guide precision medicine, advancing mechanistic interpretation of the disease and supporting more effective, patient‐tailored intervention.

Despite the increasing adoption of targeted‐immunotherapy combinations in HCC, therapeutic resistance remains a major hurdle. As discussed above, resistance is often driven by pathway crosstalk and remodelling of the TME. Future research should explore the potential of combining targeted therapies and immunotherapies with inhibitors targeting other pathways, such as the PI3K and Hh pathways, to address the drug resistance. However, the expression of these pathways in normal tissues must also be considered to minimize off‐target effects and TRAEs. Pretreatment biopsies may help identify suitable patients for such combination therapies, thereby reducing unnecessary adverse reactions.

While current first‐line treatment strategies primarily focus on combination therapies, second‐line treatments are still predominantly based on monotherapy. The role of combination therapies in second‐line and subsequent treatments warrants further exploration. In addition to the aforementioned areas of future research, it is important to consider the need for new therapeutic combinations and the integration of emerging technologies to overcome existing limitations in HCC treatment.

In conclusion, while targeted therapies and immunotherapies have shown promise in HCC treatment, there is still much work to be done. Continued advancements in biomarker discovery, biopsy‐guided treatment decisions, and the exploration of combination therapies will be essential for improving outcomes and reducing treatment‐related complications in patients with HCC.

## Author Contributions

Penghui He and Sinan Xie: conceptualization, literature search, data curation, and writing – original draft. Kunlin Xie, Fengwei Gao, Yunshi Cai, and Yan Huang: literature search, writing – review and editing, and visualization. Yinghao Lyu, Hong Wu, and Tian Lan: conceptualization, supervision, writing – review and editing, and funding acquisition. All authors have read and agreed to the published version of the manuscript.

## Funding

The study was supported by grants from the National Natural Science Foundation of China (82573497, 82372791 and 82303220), the National Major Science and Technology Projects of China (2023ZD0502002), the Science and Technology Support Program of Sichuan Province (2024NSFSC0745, 2025ZNSFSC1900, 2025ZNSFSC1901 and 2026NSFSC1950), the National Postdoctoral Researcher Program of China (GZC20251423), West China Hospital of Sichuan University Postdoctoral Research Development Fund (2025HXBH103) and Peak Discipline Operation Fund of West China Hospital (GFYX25004).

## Ethics Statement

The authors have nothing to report.

## Conflicts of Interest

The authors declare no conflicts of interest.

## Data Availability

The authors have nothing to report.
